# Recent advances in targeted antibacterial therapy basing on nanomaterials

**DOI:** 10.1002/EXP.20210117

**Published:** 2023-02-05

**Authors:** Zhongmin Geng, Zhenping Cao, Jinyao Liu

**Affiliations:** ^1^ Shanghai Key Laboratory for Nucleic Acid Chemistry and Nanomedicine, Institute of Molecular Medicine, State Key Laboratory of Oncogenes and Related Genes, Shanghai Cancer Institute, Renji Hospital, School of Medicine Shanghai Jiao Tong University Shanghai China; ^2^ The Affiliated Hospital of Qingdao University Qingdao University Qingdao China; ^3^ Qingdao Cancer Institute Qingdao University Qingdao China

**Keywords:** antibacterial, drug delivery, nanocarriers, nanomaterials, targeted therapy

## Abstract

Bacterial infection has become one of the leading causes of death worldwide, particularly in low‐income countries. Despite the fact that antibiotics have provided successful management in bacterial infections, the long‐term overconsumption and abuse of antibiotics has contributed to the emergence of multidrug resistant bacteria. To address this challenge, nanomaterials with intrinsic antibacterial properties or that serve as drug carriers have been substantially developed as an alternative to fight against bacterial infection. Systematically and deeply understanding the antibacterial mechanisms of nanomaterials is extremely important for designing new therapeutics. Recently, nanomaterials‐mediated targeted bacteria depletion in either a passive or active manner is one of the most promising approaches for antibacterial treatment by increasing local concentration around bacterial cells to enhance inhibitory activity and reduce side effects. Passive targeting approach is widely explored by searching nanomaterial‐based alternatives to antibiotics, while active targeting strategy relies on biomimetic or biomolecular surface feature that can selectively recognize targeted bacteria. In this review article, we summarize the recent developments in the field of targeted antibacterial therapy based on nanomaterials, which will promote more innovative thinking focusing on the treatment of multidrug‐resistant bacteria.

## Graphical abstract

To address the challenge of antibiotic‐resistant bacteria, nanomaterials‐mediated targeted bacteria depletion in either a passive or active manner has emerged as one of the most promising approaches for antibacterial treatment. In this review, we summarize the recent development in the field of targeted antibacterial therapy based on nanomaterials, which show advantages in increasing local concentration around bacterial cells to enhance inhibitory activity and reduce side effects. This work would promote more innovative thinking focusing on the treatment of multidrug‐resistant bacteria.

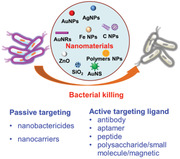

## 1 INTRODUCTION

According to the report of the World Health Organization, bacterial infection is one of the leading causes of death worldwide in the past 15 years.^[^ ^1^ ^]^ Although antibiotics as conventional antibacterial drugs have achieved huge success in treating bacterial infectious diseases, extensive and inappropriate consumption of antibiotics leads to the occurrence of multidrug resistance.^[^ ^2^, ^3^ ^]^ As it threatens human beings’ health at any stage of life via food, water, and livestock,^[^ ^4^, ^5^ ^]^ antibiotic resistance has become one of the most urgent public health problems in the world. It is reported that each year nearly 3 million people are infected with antibiotic‐resistant bacteria and at least 35,000 deaths are caused as a direct consequence of these infections, resulting in an estimated cost of infection treatment of ∼20 billion United States dollar (USD) annually in the United States.^[^ ^6^, ^7^ ^]^ Therefore, there remains an urgent need to develop new antibacterial agents with low side effects and high efficacy.^[^ ^8^ ^]^

To tackle this challenge, various long‐term approaches have been utilized to propose viable options against antibiotic‐resistant bacterial infection,^[^ ^9^, ^10^, ^11^ ^]^ such as the discovery of new inhibitors,^[^ ^11^, ^12^, ^13^ ^]^ the interference of quorum sensing,^[^ ^14^, ^15^ ^]^ and the development of new antibiotics.^[^ ^16^, ^17^, ^18^, ^19^ ^]^ In the meantime, significant and intensive efforts have been invested in short‐term approaches, such as chemically modifying existing antibiotics for drug resistance elimination. However, neither the discovery of novel antibiotics nor the chemical modification of existing drugs, their low target selectivity results in the removal of a great deal of potent drugs from the list of clinical therapeutics.^[^ ^20^ ^]^ One promising way to address this shortcoming is rational functionalization of antibacterial agents by improving drug selectivity and targeting ability, such as conjugating antibiotics with targeting ligands that have affinity to bacterial cells.^[^ ^21^ ^]^ Targeting capability originated from conjugated specific ligands that can recognize biomolecules or receptors overexpressed on the surface of bacterial cells or infected tissues provides strongly enhanced interactions between antibiotics and bacterial cells. For example, Vitamin B9, one the of small molecules targeting ligands, has been intensively applied for clinical treatment.^[^ ^22^ ^]^ The application of targeting ligands displays many advantages, such as strong binding affinity, high specificity, intrinsic stability, and commercial availability.^[^ ^23^ ^]^ These targeted systems display that the local concentrations of antibacterial agents significantly increase at targeted sites in contrast to off‐target sites. In recent years, representative targeting ligands, such as antibodies, aptamers, and peptides, have been intensively utilized, exhibiting superior inhibitory activities against bacteria.^[^ ^3^ ^]^

**FIGURE 1:**
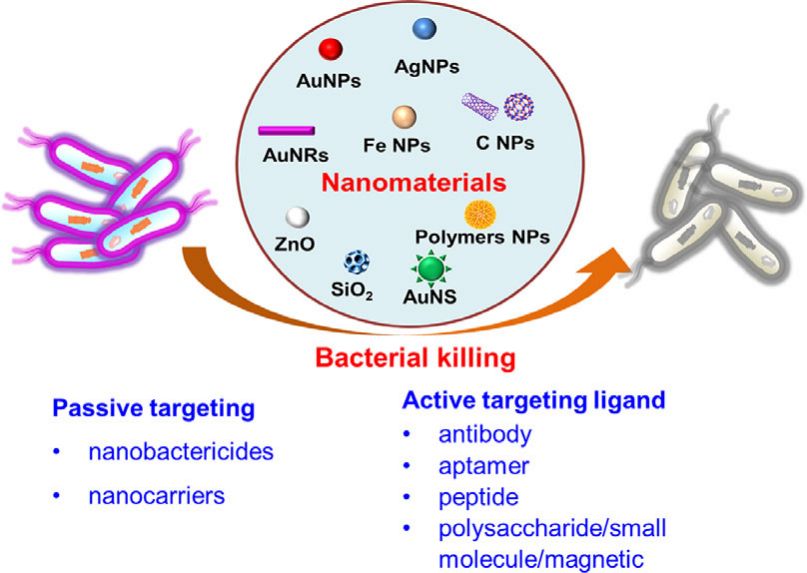
Antibacterial mechanism and nanomaterial‐mediated antibacterial therapy based on passively or actively targeting strategy

Over the last decade, nanotechnology has attracted remarkable attention in antibacterial application, showing great potential to enhance the effectiveness of antibiotic‐resistant bacteria treatment.^[^ ^24^, ^25^, ^26^ ^]^ Antibacterial abilities of nanomaterials are attributed to improve the potentiality of existing antibiotics or exert antibacterial action by nanomaterials without antibiotics.^[^ ^27^ ^]^ There are plenty of unique advantages of nanomaterials for antibacterial applications especially in combatting antibiotic‐resistant bacteria. First, nanomaterials, which can be used as antibiotic carriers, are of great help in increasing drug accumulation, reducing overall drug exposure, facilitating bacterial uptake, and improving drug stability.^[^ ^28^ ^]^ These antibiotic carriers can overcome the barrier of the cell membrane and deliver antibiotics to kill antibiotic‐resistant bacteria. Besides, multiple antibiotics can be delivered by nanomaterials and controllably released to inhibit antibiotic‐resistant bacteria. Second, their new bactericidal pathways that are totally different from antibiotics have been illustrated. As demonstrated by recent studies, antibacterial abilities of these nanomaterials are closely associated with the damages of bacterial cell membranes, which are caused by non‐oxidative bactericidal mechanisms, the release of metal ions, and the generation of reactive oxygen species (ROS).^[^ ^29^, ^30^, ^31^, ^32^, ^33^, ^34^ ^]^ Third, the physical properties of nanomaterials, including shape, size, and structure, can be easily designed and controlled. Last but not least, the surface of nanomaterials can be modified and functionalized on demand, enabling multiple antibacterial capabilities. Therefore, it is highly important to understand the intrinsic properties and antibacterial mechanisms for developing novel, effective antibacterial strategies based on these advanced characteristics of nanomaterials. In this review, we review and discuss nanomaterial‐based targeted antibacterial strategies in a passive or active fashion, by which the local concentration of nanomaterials at infection sites or around bacteria cells can be accumulated efficiently to strengthen bacteria killing (Figure 1). We also summarize the recent advance of targeted antibacterial therapy based on newly‐developed nanomaterials.

## 2 MECHANISMS OF NANOMATERIAL‐BASED ANTIBACTERIAL STRATEGIES

**FIGURE 2:**
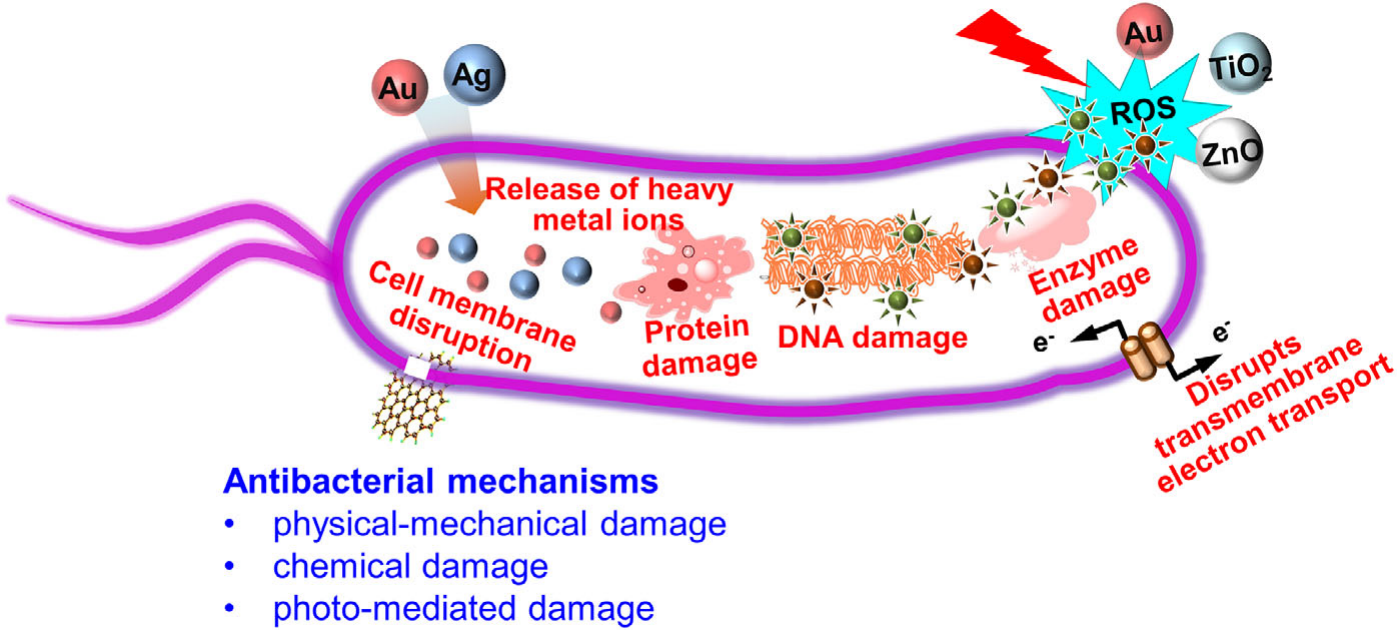
Inhibition of bacteria basing on nanomaterials by physical‐mechanical, chemical, or photo‐mediated damage

In contrast to traditional small molecular antibiotics, nanomaterials allow large amounts of molecules to attach bacterial cells through self‐assembly, resulting in different antibacterial mechanisms (Figure 2). Nanomaterials show numerous antibacterial behaviors for fighting against bacterial infection without causing antibiotic resistance,^[^ ^35^, ^36^ ^]^ including physical‐mechanical, chemical, and photo‐mediated damages.^[^ ^37^ ^]^ Therefore, understanding the interactions between nanomaterials and bacterial cells can contribute to a rational design of potent nanotherapeutics with the possibility to replace conventional antibiotics in antibacterial treatment.

### 2.1 Bacterial inhibition based on physical‐mechanical damage of cellular membranes

Bacterial cell membrane that plays essential roles in keeping bacteria intact and alive is considered as one of the most important antibacterial targets. Similar to conventional antibiotics, nanomaterials with unique physiochemical properties including size, surface charge, and topological structure can cause damages to bacterial cell membrane after direct contact with bacteria.^[^ ^38^, ^39^ ^]^ It has been reported that topological nanostructures mimicking the surface of living organisms possess antibacterial ability.^[^ ^40^ ^]^ Crawford et al. have reported that cicada wing surface displays effective antibacterial properties via physical‐mechanical effect.^[^ ^41^ ^]^ Additionally, the sharp edge of nanoparticles (NPs) can damage bacterial cell membrane. Li et al. have proved that the sharp corners of graphene nanosheets penetrate into bacteria along with inducing cell wall damage.^[^ ^42^ ^]^ Recently, the hydrophobic and electrical properties of nanomaterials have been verified to cause bacterial damage by breaking bacterial cell wall.^[^ ^43^ ^]^

### 2.2 Chemical damage‐induced bacterial killing

Different from physical‐mechanical damage to bacterial cells, chemical damage is mainly caused by generating oxidative stress, which could damage proteins or genes.^[^ ^44^ ^]^ Numerous attempts have been made to develop antibacterial nanomaterials with efficient ROS generation. Wang et al. have successfully developed a new type of zwitterionic polymer nanomicelles with enhanced ROS generation for photodynamic antimicrobial therapy, owing to the presence of an aggregation‐induced emission active photosensitizer moiety.^[^ ^45^ ^]^ The ROS generation efficiency of this nano‐system was evaluated by a ROS‐sensitive probe, which exhibited a 5.8‐time increment in fluorescence intensity within 30 s under light irradiation. Reactive nitrogen species, another class of chemically reactive species, can be produced by nanomaterials for inhibiting the growth of bacteria via nitrosative stress.^[^ ^46^, ^47^ ^]^ Due to the short half‐life of free radical nitric oxide (NO) and their derivatives, several NO‐donor combined nanomaterials, including dendrimers,^[^ ^48^ ^]^ NPs,^[^ ^49^ ^]^ and polymers,^[^ ^50^ ^]^ have been developed for stabilizing NO and extending their half‐life to kill bacteria. Seabra et al. have exploited a chemically modified chitosan approach to obtain S‐nitroso‐chitosan for antibacterial applications.^[^ ^51^ ^]^ S‐nitroso‐chitosan can release NO sustainably at a steady‐state rate of 42.2 ± 0.7 mmol L^−1^ to achieve long‐term antibacterial effects.

### 2.3 Photo‐mediated bacterial killing

**FIGURE 3:**
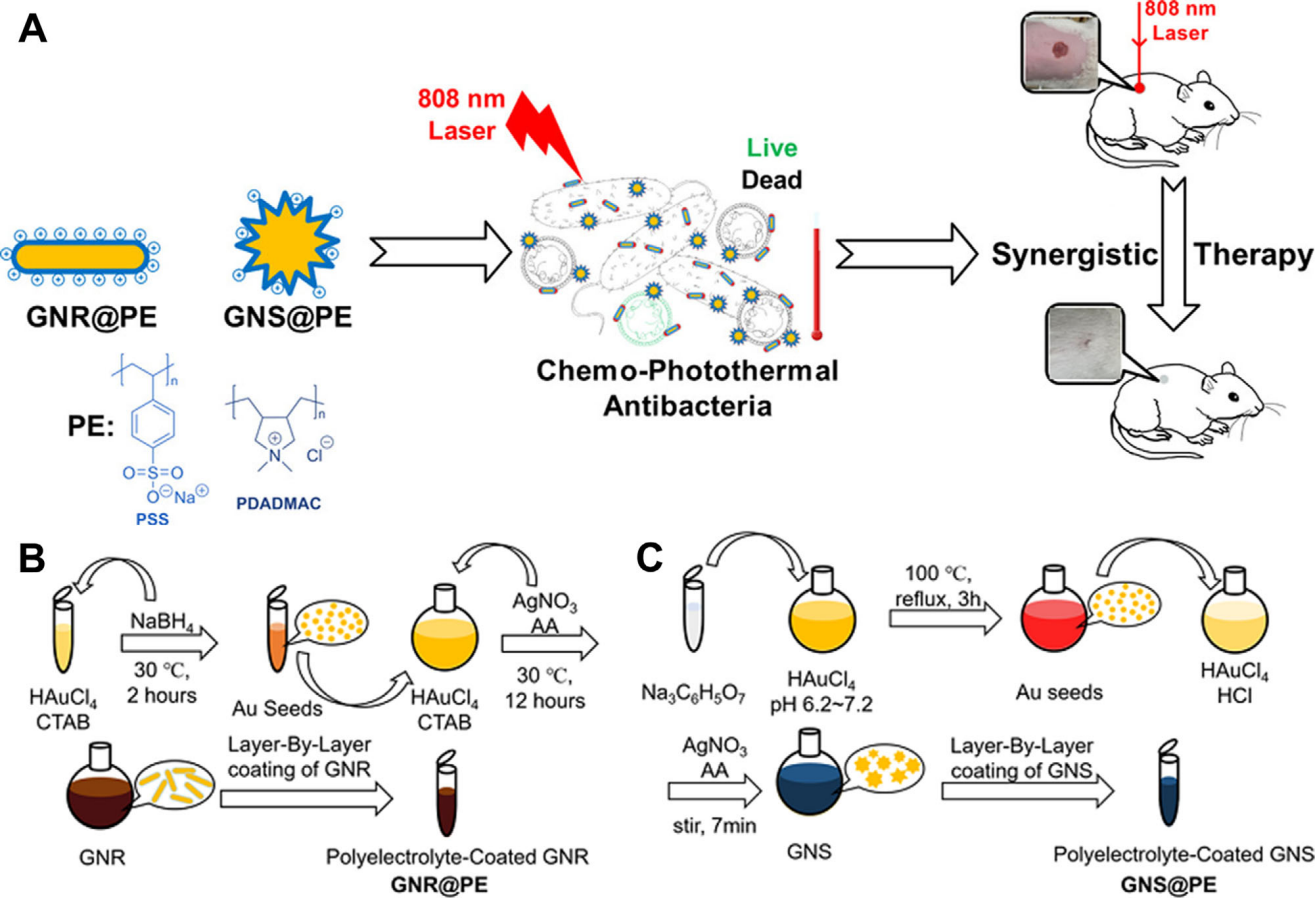
(A) Schematic depiction of synthesis and application of GNR@PE and GNS@PE, respectively. Illustration of coating processes of (B) PE‐coated GNR and (C) PE‐coated GNS. Reproduced with permission from.^[^ ^54^ ^]^ Copyright 2020, American Chemical Society

Photothermal therapy has emerged as a promising strategy for antibacterial treatment by converting light into heat under the irradiation of near‐infrared (NIR) or visible light. It has been reported that a variety of photothermal nanomaterials, such as carbon‐based nanocomposites, noble metallic nanomaterials, as well as polymeric nanomaterials and metal sulfide nanomaterials, have displayed high light‐to‐thermal conversion efficiencies.^[^ ^52^, ^53^ ^]^ Surface coating is a flexible and effective strategy to achieve enhanced photothermal therapy efficacy. For instance, Wang et al. have designed polyelectrolyte‐coated gold nanomaterials (GNR@PE and GNS@PE) to obtain an antibacterial effect synergistically (Figure 3).^[^ ^54^ ^]^ GNR@PE and GNS@PE demonstrate great synergistic chemo‐photothermal antibacterial efficiency in a mouse wound infection model under the irradiation of an 808 nm laser. To enhance photothermal therapy efficacy, combination with one or two other approaches has been applied in antibacterial treatments. For example, Deng et al. have successfully synthesized multifunctional, ultrasmall‐sized gold‐platinum nanodots which are profited from the combination of photothermal and chemodynamic therapy.^[^ ^55^ ^]^ Patir et al. have reported a new black phosphorus/Au nanocomposite, which exhibits highly antibacterial efficiency due to the combination of photothermal antibacterial and antibiofilm effects.^[^ ^56^ ^]^

Photodynamic therapy (PDT) is exploited as an alternative antibacterial therapeutic modality to control the spread of resistant bacteria.^[^ ^57^ ^]^ Different from traditional antibiotics that bind to a single molecular target, ROS can interact with multiple sites on bacteria to gain more opportunities for bacterial inactivation. Metal‐ and carbon‐based nanomaterials have been proven to remarkably enhance the antibacterial efficacy of PDT.^[^ ^58^, ^59^ ^]^ In addition, sunlight‐triggered nanomaterials have also been applied for antibacterial treatment by solar light‐induced ROS generation. Although studies of sunlight‐triggered nanomaterials are limited, with the development of visible photocatalysts, sunlight‐enabled antibacterial nanomaterials will attract increasing attention.^[^ ^60^, ^61^ ^]^

## 3 NANOMATERIALS WITH PASSIVE TARGETING ANTIBACTERIAL CAPABILITIES

**FIGURE 4:**
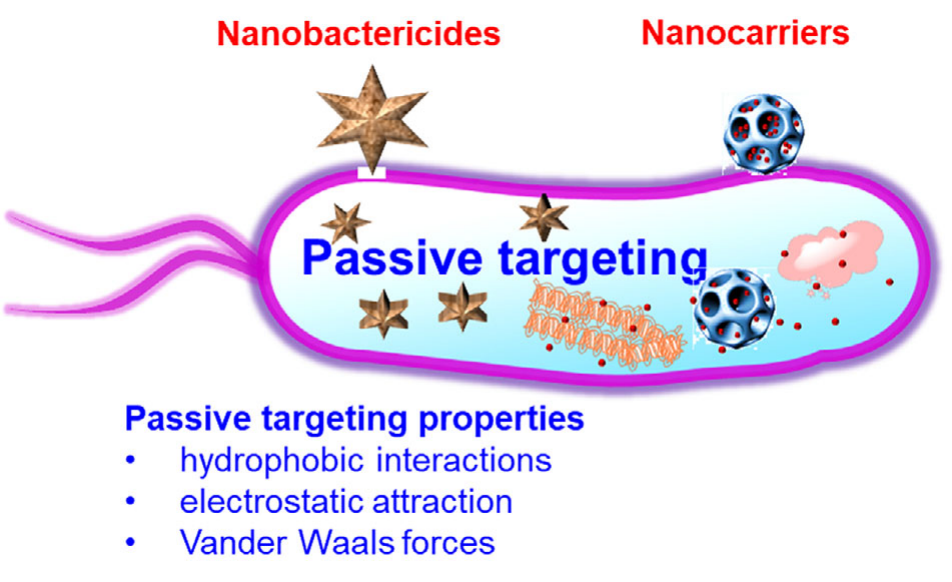
Antibacterial nanomaterials and their applications for antibacterial therapy in a passive targeting manner

Nanomaterials are designed to transport to target sites in a passive or active delivery route, which is mediated by instinctive reaction, targeting moiety, and external force.^[^ ^62^ ^]^ Passive targeting, as an efficient method to deliver drug to bacteria, occurs to modified nanomaterials that arrive at target tissues through diffusion or convection (Figure 4).^[^ ^63^ ^]^ Investigations into the antibacterial mechanism of nanomaterials have revealed that nanomaterials could interact with key components of the bacterial cell membrane such as lipids, proteins, and polysaccharides through hydrophobic interactions, electrostatic attraction, and Vander Waals forces.^[64,65^^]^ For example, the antibacterial action of AuNPs/AgNPs was attributed to multiple types of interactions (such as electrostatic attraction and hydrophobic interactions) between NPs and bacterial proteins.^[^ ^66^ ^]^ In general, the physicochemical properties of the bacterial cell membrane and nanomaterials directly determine the interactions and antibacterial effects. Although bacteria have evolved numerous strategies to circumvent the damaging effects of antibiotics, it is difficult for bacterial cells to develop resistance to nanomaterials that could kill bacteria in multiple ways at the same time.^[^ ^25^ ^]^ With the development of nanotechnology, different sizes and forms of nanomaterials, including nanotubes,^[67,^ ^67^, ^68^ ^]^ nanofibers,^[69,^ ^69^, ^70^ ^]^ NPs, and nanocomposites,^[71,^ ^71^, ^72^ ^]^ have been applied in the diagnosis and treatment of various kinds of bacterial infections.

### 3.1 Nanobactericides

**FIGURE 5:**
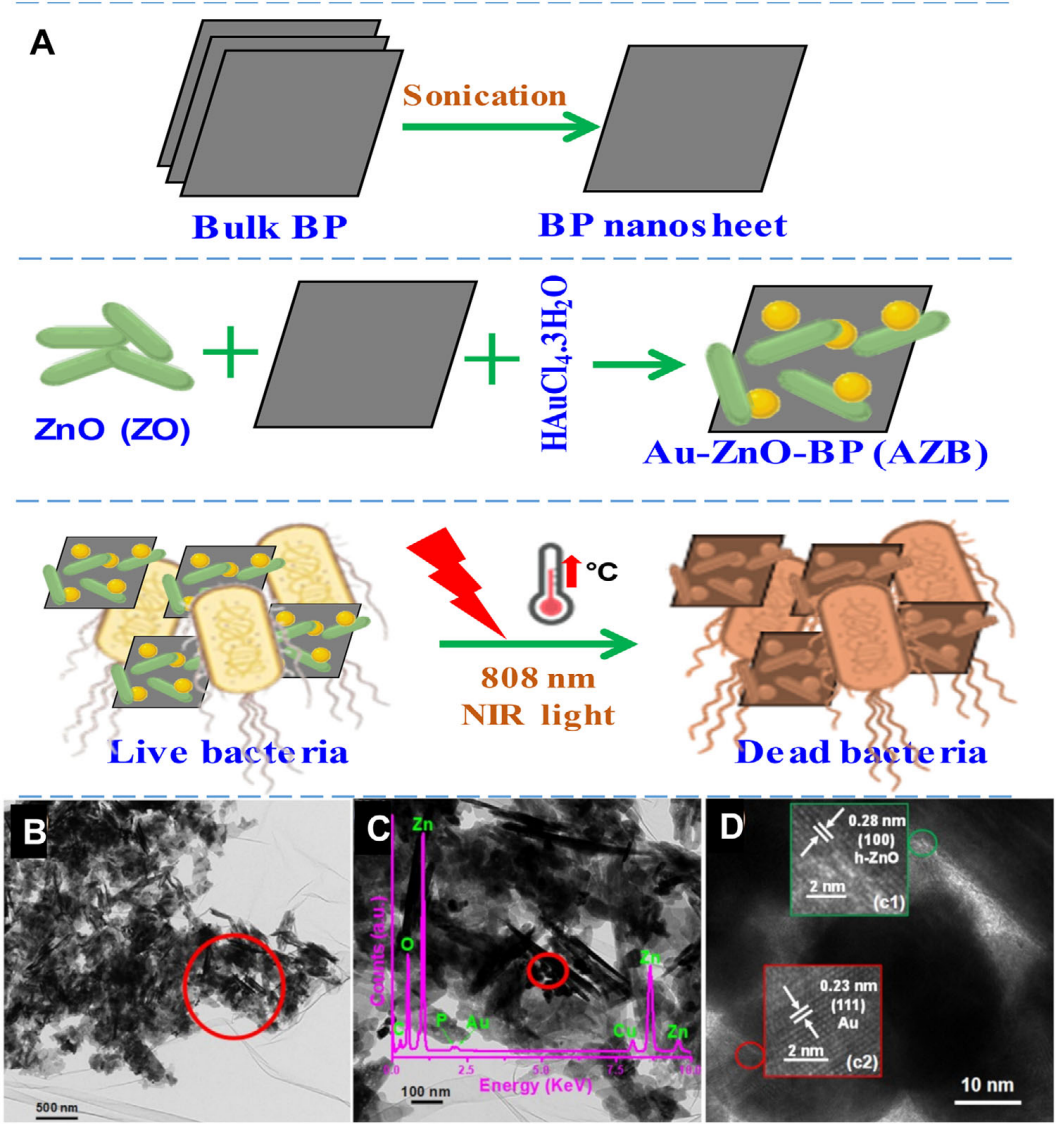
(A) Preparation and application of Au‐ZnO‐BP nanoantibiotic as a synergistic antibacterial agent against *S. aureus* species. (B) A typical transmission electron microscopy (TEM) image of AZB sample. (C) An enlarged TEM image of AZB sample and corresponding energy‐dispersive X‐ray spectrum. (D) A high‐resolution TEM image presenting ZO and Au NPs. Reproduced with permission from.^[^ ^79^ ^]^ Copyright 2021, MDPI

NPs, which display antibacterial activity by themselves or improve the effectiveness of antibiotics, are termed as nanobactericides. Typically, but not exclusively, metals, metal oxides, and carbon‐based NPs display antibiotic activity naturally via multiple mechanisms, such as inhibiting the activity of enzymes, interrupting DNA synthesis, producing ROS, and hindering energy transduction.^[73,^ ^73^, ^74^ ^]^ As a result, a great number of nanomaterials have been exploited for inhibiting the growth of bacteria, including silver NPs (AgNPs), zinc oxide NPs, titanium dioxide NPs, gold NPs (AuNPs), aluminum NPs, copper NPs, carbon nanomaterials, iron NPs, and chitosan NPs. It has been reported that NPs can easily enter bacterial cells through bacterial cell pores by passive diffusion.^[75,^ ^75^, ^76^ ^]^ Passive targeting based on the modulation of nanomaterial structure and physico‐chemical properties is an efficient method for antibacterial treatment. Recently, Cheon et al. have proposed that the antibacterial activity of NPs depends on their morphology, which can optimize Ag ion release and target delivery.^[^ ^77^ ^]^ To prove this hypothesis, AgNPs with spherical, triangular plate, and disk morphologies are synthesized and their antimicrobial activities are examined using a disk diffusion method against *S. aureus*, *E. coli*, and *Pseudomonas aeruginosa*. The results demonstrate that spherical AgNPs show the highest inhibition activity against *E. coli*, given that the different surface areas of spherical, triangular plate, and disk shapes can lead to varied release concentrations of Ag ions. This morphology‐dependent antibacterial property suggests a multifaceted antibacterial profile of NPs. Meanwhile, the results prove that the shape of NPs is an important parameter for antibacterial activity, beside the size of NPs. NIR light‐mediated photothermal therapy has been widely applied in cancer therapy due to its relative deep tissue penetration and few side effects.^[^ ^78^ ^]^ Similarly, it is explored as a promising candidate antibacterial strategy for inhibiting bacterial growth. Kim et al. have prepared a NIR‐assisted black phosphorus conjugated with ZnO and Au (Au‐ZO‐BP) to inhibit *S. aureus* species (Figure 5).^[^ ^79^ ^]^ Black phosphorus, a rising star of two‐dimensional biomedical nanomaterials, has received increasing attention due to its superior NIR absorption property. For example, black phosphorus nanosheets containing AuNPs have been prepared to assemble in situ with ZnO at a low temperature condition. The antibacterial activity of Au‐ZO‐BP nanocomposite is investigated by incubating with non‐, erythromycin‐, and methicillin‐resistant *S. aureus* (MRSA) species plus NIR irradiation. The results indicate that this nanocomposite shows excellent anti‐*S. aureus* ability as well as low antibiotic resistance after a long‐term treatment. Despite black phosphorus based Au‐ZO‐BP nanocomposite displays enormous biomedical potential, the knowledge of interactions between the protein and black phosphorus is still limited.

Previous preparation of metal‐based nanobactericides typically needs various types of chemicals at high temperatures, which is not an environment‐friendly synthesis method. In recent years, green synthesis of metal NPs and their oxides, which is eco‐friendly, safe, affordable, and cost‐effective by avoiding the use of expensive, harsh, and toxic raw materials, has been widely implemented.^[^ ^80^, ^81^, ^82^ ^]^ For instance, noble metal‐based NPs have been synthesized from plant extracts. Kirakosyan et al. have synthesized AgNPs using extracts of *Agastache foeniculum* plants and callus, showing near‐spherical shape and extract source‐dependent particle size.^[^ ^83^ ^]^ Their antibacterial activities have been assessed by a few pathogenic bacteria for hospital‐acquired infections, including *S. aureus*, *S. haemolyticus*, *Streptococcus pneumonia*, and *Klebsiella pneumonia* and the results exhibit that AgNPs can effectively inhibit the growth of all the tested strains at a low particle concentration. Interestingly, the antibacterial activity of these AgNPs is remarkably higher than that of AgNPs that are synthesized similarly in previous works, suggesting a dependence on preparation conditions. Furthermore, Mousavi et al. have evaluated the antibacterial efficiency of green iron nanoparticles (FeNPs), which are prepared using *Satureja hortensis* essential oil.^[^ ^84^ ^]^ Their antibacterial efficacies against Gram‐positive bacteria (*S. aureus* and *Corynebacterium glutamicum*), Gram‐negative bacteria (*P. aeruginosa* and *E. coli*), and one fungus species *Candida albicans* are evaluated, and the results verify impressively high inhibition efficiency, especially for Gram‐negative bacteria. Thovhogi et al. have reported a new type of copper oxide nanoparticles (CuO NPs) obtained from an extract of Cedrus deodara by using facile, nontoxic, and low‐cost green synthesis.^[^ ^85^ ^]^ The superior antibacterial and antiproliferative potency of these biosynthesized CuO NPs is confirmed by using *E. coli* and *S. aureus*. MgO NPs synthesized by Simanjuntak et al. via using *Moringa oleifera* leaf water extracts and a solution of magnesium chloride exhibit remarkable antibacterial property.^[^ ^86^ ^]^ Similarly, Gedanken et al. have successfully synthesized carbon dots using medicinal turmeric leaves (*Curcuma longa*), showing favorable antibacterial activity against both Gram‐negative bacteria and gram‐positive bacteria.^[^ ^87^ ^]^ Thus, as we can see, the biological synthesis of nanobactericides has attracted more and more attention due to their advantages of being environment‐friendly, nontoxic, and low‐cost. Although the biological synthesis process reduces the toxicity, the role of plant extracts in the synthesis and activity of NPs has not been clearly understood. Synthesis using pure compounds extracted from plant extracts may provide additional effective solutions regarding the disparity of green NPs synthesis.

### 3.2 Nanocarriers

Continual advances in nanotechnology promote the development of drug delivery systems that are used for antibiotic transport with enhanced drug accumulation and reduced side effects. The enhanced permeability and retention (EPR) effects have been mainly considered as a basic route for not only passive drug targeting to tumor site, but also for infection and inflammation lesions.^[^ ^88^ ^]^ Taking advantage of EPR effects at infection sites, nanotherapeutic approaches have been exploited to manage antibacterial agents.^[89^^]^ In this regard, nanocarriers as efficient antibacterial drug delivery systems show several merits. First, nanocarriers can be designed with stimuli‐responsiveness for targeted drug delivery. Second, antibacterial drugs based on nanocarriers can be endowed with a significantly extended half‐life during blood circulation, controlled drug release, and optimized pharmacokinetics. Moreover, thanks to their unique physicochemical properties, nanocarriers can be easily tailored to minimize side effects and increase drug loading. Last, nanocarriers can improve antibacterial drugs’ solubility and stability and load multiple drugs for synergistic antibacterial therapy.

Various types of nanocarriers, such as polymeric nanomicelles, dendrimers, metallic NPs, and liposomes, have been investigated for delivering antibiotics.^[90^^]^ Due to the multiple drug‐loading capabilities of nanocarriers, Fang et al. have designed a synergistic therapy strategy with a dual antibacterial and anti‑inflammatory effect to treat bacterial infection.^[^ ^91^ ^]^ In this study, the authors have developed nanostructured lipid carriers (NLCs), which can load ciprofloxacin and rolipram simultaneously. In vitro tests of antibacterial and anti‐inflammatory abilities reveals superior effects in comparison with the combination of free drugs. Further, in vivo treatment with NLCs displays improvements in bacterial clearance and the prevention of organ damage in MRSA‐infected mice. Given specific targeting ability and drug release profile, stimuli‐responsive nanocarriers have received increased attentions worldwide. Govender et al. have reported a combined strategy to improve the potency of antibiotics against infectious diseases using pH‐responsive nanosystems that can co‐deliver vancomycin and 18β‐glycyrrhetinic acid (VCM‐GAPAH‐LPHNPs).^[^ ^92^ ^]^ In vitro evaluation has confirmed the enhanced biocompatibility and biosafety of VCM‐GAPAH‐LPHNPs. Compared to free vancomycin and 18β‐glycyrrhetinic acid, treatment with VCM‐GAPAH‐LPHNPs in an acidic medium can improve antibacterial efficacy against MRSA. Peptide based controllable self‐assemblies have been developed as a promising candidate for antibacterial application. Wang et al. have reported a sandwich dimeric structure that was self‐assembled by peptide‐chlorophyll conjugates with Cu^2+^.^[^ ^93^ ^]^ This dimer could target macrophages and significantly eliminate intracellular *S. aureus* in mice muscular infection model by PDT. Very recently, they have successfully applied human defensin‐6 peptide to self‐assemble into nanoparticles, which could trap bacteria and prevent bacterial invasion.^[^ ^94^ ^]^ Self‐disassembled nanovesicles have been designed by the Wang group for precise oritavancin antibiotic delivery and enhanced antibacterial efficiency.^[^ ^95^ ^]^ Considering the complex microenvironment of the infected sites, nanocarriers with individual stimulus‐responsiveness are sometimes insufficient for delivering antibacterial drugs. To address this issue, Wang et al. have designed and prepared smart dual‐responsive cellulose nanofibers (CNF‐PEI‐NIPAM), presenting notable biocompatibility and antibacterial activity during in vitro and in vivo evaluations.^[^ ^96^ ^]^ The antibacterial activity of CNF‐PEI‐NIPAM was above 99% against *E. coli*, due to the high density of amino groups. To date, a large number of nanobactericides and nanocarriers have been applied to upgrade the delivery of antibiotics. Compared to free antibiotics, considerable progress of NPs has been achieved in antibacterial therapy, including targeted delivery, efficient drug loading, overcoming resistance, and protection from inactivation. Although nanocarriers have promising potential for bacterial killing, the study of nanocarriers is still in the preliminary stage of testing. More studies of the interactions between nanocarriers and biological molecules are needed to ensure their biosafety and efficacy in vivo.

## 4 NANOMATERIALS WITH ACTIVE TARGETING ANTIBACTERIAL EFFECTS

**FIGURE 6:**
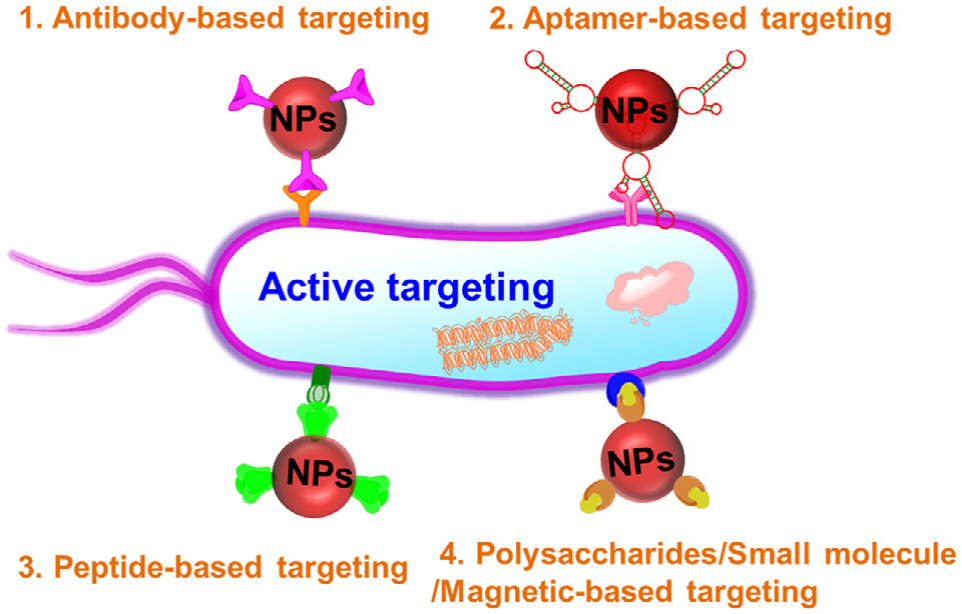
Nanomaterials with surface conjugation of antibodies, aptamers, peptides, polysaccharides, or small molecular ligands for antibacterial treatment in an active targeting fashion

Despite its elegance, passive targeting strategy often suffers from a few limitations, including the dependence on circulation time for drug accumulation in target tissues, weak specificity, and the random nature of the delivery process (interaction between NPs and cells is driven by stochastic processes).^[97,^ ^97^, ^98^ ^]^ To overcome these limitations, active targeting strategy has been emerged and broadly utilized antibacterial therapy. Active targeting involves the application of specific ligands that can recognize target structures/substrates for enhanced delivery of antimicrobial agents to bacterial cells (Figure 6).^[99^^]^ Owing to the characteristics of nanoscale size and surface properties, the half‐life of nanomaterials can be significantly extended in blood circulation, resulting in an increment in the local concentration of therapeutics at infection sites.^[^ ^100^ ^]^ Ligands with affinity for molecules or receptors on cell surfaces boost cellular uptake of nanomaterials and lead to an improved therapeutic effect.^[^ ^101^ ^]^ The surface of nanomaterials is typically conjugated with targeting ligands, such as antibodies, aptamers, peptides, polysaccharides, and specific small molecules, to selectively recognize bacterial cells.

### 4.1 Antibody‐conjugated nanomaterials

Antibodies have been highly pursued in basic research, benefiting from high specificity and affinity for target antigens. However, the low therapeutic activity of antibodies has limited their wide application in antibacterial treatment.^[^ ^102^ ^]^ In the last twenty years, researchers have found that antibodies conjugated with nanomaterials can strikingly enhance therapeutic potency. Thus, the antibody conjugation strategy has become increasingly attractive for the treatment of both inflammatory diseases and cancers.^[103‐105^^]^ A wide variety of nanomaterials, such as metal NPs, polymeric NPs, carbon nanotubes, and metal oxide NPs, have been conjugated with antibodies for antibacterial therapy.

Given the optical and photothermal properties, gold nanomaterials with different sizes and shapes have been conjugated with antibodies for killing bacterial cells.^[106,^ ^106^, ^107^ ^]^ Norman et al. have developed a new approach by using antibody‐conjugated gold nanorods to selectively target and destroy bacterial cells.^[108^^]^ Gold nanorods that are covalently anchored with antibodies can enhance their binding with the surface of *P. aeruginosa*, resulting in a significant reduction in cell viability after NIR radiation. Wang et al. have conjugated anti‐*Salmonella* antibody to oval‐shaped AuNPs for label‐free detection and destruction of *S. typhimurium*.^[109^^]^ These antibody‐conjugated AuNPs can bind with cells to form microbial clusters, resulting in irreparable cellular damage after exposure to NIR irradiation. Moreover, different structures and morphologies of gold nanomaterials lead to tunable optical/plasmonic properties under laser irradiation.^[^ ^110^ ^]^ Recently, Zharov et al. have used 10‐, 20‐, and 40‐nm AuNPs that were conjugated with anti‐*S. aureus* antibodies to target the bacterial wall for selective killing of *S. aureus*.^[^ ^111^ ^]^ This study has successfully optimized the size‐dependent efficiency of AuNPs in bacterial inhibition. Meanwhile, this approach can regulate the attachment efficiency of antibody‐conjugated NPs to bacterial cell surfaces by controlling the number of conjugated antibodies. Although antibody‐conjugated AuNPs have been widely implemented in antibacterial therapy, significantly reduced efficacy has frequently occurred in biofilm‐associated infections. Meeker et al. have explored an antibody‐conjugated gold nanocage coated with antibiotic‐loaded polydopamine (PDA).^[^ ^112^ ^]^ Upon laser irradiation, this system can be activated to convert photon energy into heating, leading to controlled release of antibiotics in infection tissues. This work proposes that the use of photothermal effects to synergize antibiotic delivery promotes treatment efficiency toward bacterial infections. It highlights the feasibility of incorporating alternative antibodies and antibiotics to PDA‐coated gold nanocages for targeting *P. aeruginosa*.^[^ ^113^ ^]^ Impressively, this approach has been confirmed to achieve enhanced laser‐assisted photothermal treatment and antibiotic‐mediated killing of both Gram‐positive and Gram‐negative bacterial species.

Various types of carbon‐based nanostructures have been used for antibacterial agents, due to their environment‐friendly and high bacterial toxic properties.^[52^ ^52^, ^114^ ^]^ Particularly, a series of carbon nanostructures, including graphene oxide, carbon colloids, and carbon nanotubes, have attracted considerable attention in recent years. Reid et al. have applied anti‐*Streptococcus* group A antibody‐conjugated multi‐walled carbon nanotubes to investigate targeted photothermal damage of both planktonic bacteria and biofilm‐residing bacteria.^[^ ^115^ ^]^ Moreover, the authors have found that light‐induced local heating causes negligible collateral damage in adjacent tissue, which supports the potential of carbon nanotubes for further translation. Chen et al. have used antibody‐functionalized nanoscale reduced graphene oxide (NRGO) for selective killing of *S. aureus*, showing negligible toxicity to *E. coli* and human cells under NIR irradiation.^[^ ^116^ ^]^ These easy‐fabricated and cost‐effective properties support the use of antibody‐NRGO as a promising candidate for antibacterial therapy. More recently, Hamme et al. have fabricated both anti‐*E. coli* monoclonal antibody‐conjugated and gold nanopopcorns attached single‐walled carbon nanotubes (mAb‐AuNP@f_3_‐SWCNTs) for selective detection and specifical photothermal damage of bacteria.^[^ ^117^ ^]^ Under 670 nm light irradiation, mAb‐AuNP@f_3_‐SWCNTs show marked sensitivity and rapid selectivity, achieving a higher killing efficiency against *E. coli* than unmodified AuNPs under the same laser exposure.

**FIGURE 7:**
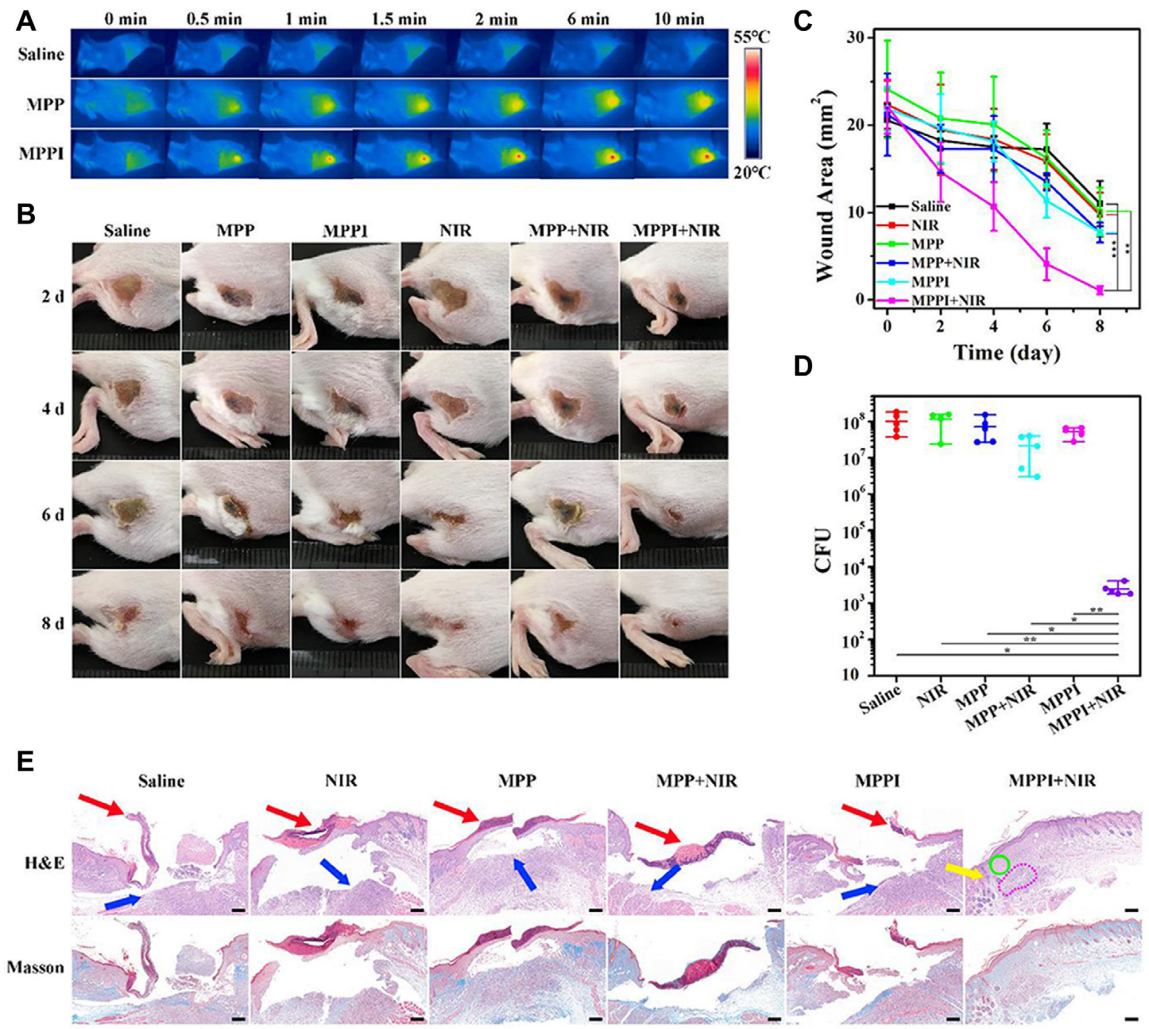
(A) IR thermal images of *S. aureus* infected tissues after different treatments. (B) Digital images of *S. aureus* infected tissues after treatment. (C) Variation of wound area after different treatments. (D) Colony‐forming units (CFU) of *S. aureus* in infected tissues 8 days post‐treatment. (E) Hematoxylin and eosin and Masson's trichrome staining of *S. aureus* infected tissues after treatment. Reproduced with permission from.^[^ ^124^ ^]^ Copyright 2019, Frontiers Media SA

In addition to gold and carbon nanomaterials, other antibody‐conjugated metal, metal oxide, polymer, and inorganic nanomaterials also have demonstrated satisfactory antibacterial activities.^[118‐122^^]^ Wang et al. have prepared anti‐*R. solanacearum* antibody functionalized PLGA NPs carrying methyl caffeate and caffeic acid phenethyl ester for selective killing of Gram‐negative *R. solanacearum*.^[^ ^12^ ^]^ The EC_50_ level of antibody‐conjugated PLGA NPs was 0.021 mg/mL, which was decreased by 92% in contrast to unmodified NPs. Therefore, the antibody‐conjugated PLGA NPs not only increase the antibacterial activity against *R. solanacearum* but also provide a new strategy for preventing and treating *R. solanacearum*. Similarly, anti‐*S. aureus* antibody‐conjugated MoS_2_ nanosheets have been successfully prepared by Wang et al. for targeted photothermal therapy of *S. aureus* infection.^[^ ^124^ ^]^ MoS_2_ nanosheets simultaneously coated with PDA and conjugated with thiol‐polyethylene glycol (PEG‐SH) and antibodies against protein A IgG (MoS_2_@PDA‐PEG/IgG NSs) can be exploited as a photoactivatable and highly selective antibacterial agent that appears to have high biocompatibility, suitable stability, photothermal property, and specific bacterial‐targeting ability (Figure 7). The obtained MoS_2_@PDA‐PEG/IgG NSs can effectively and specifically accumulate in *S. aureus* biofilm, showing a five‐times increment compared to that of MoS_2_@PDA‐PEG NSs. Furthermore, MoS_2_@PDA‐PEG/IgG NSs claim limited toxicity to normal tissues under laser irradiation, indicating acceptable safety for in vivo implementation. Karakasyan et al. have described a polymeric nanocarrier (NPs_Rif_anti‐*S. aureus*) for targeted delivery of antibiotics to *S. aureus*‐infected tissues.^[^ ^125^ ^]^ NPs_Rif_anti‐*S. aureus* are prepared by nanoprecipitation of poly(d,l‐lactic‐*co*‐glycolic acid) (PLGA) and poly(d,l‐lactic‐*co*‐glycolic acid)‐*b*‐poly(ethylene glycol) (PLGA‐*b*‐PEG), followed by conjugation with anti‐*S. aureus* antibody. As reported, NPs_Rif_anti‐*S. aureus* can specifically attach to the cell surface of *S. aureus* and then rapidly penetrate into the *S. aureus* biofilm due to the nanoscale size and presence of a PEG shell. Compared to free antibiotics (Rif), NPs_Rif_anti‐*S. aureus* display a significantly enhanced treatment efficacy in vivo, thank to actively targeted accumulation of Rif in the infected tissue. Tzanov et al. have developed an approach to promote selective destruction of targeted bacteria using bacterial‐specific antibody‐conjugated nanocapsule loaded with antibacterial essential oil (Ab@EO NCs).^[^ ^126^ ^]^ An anti‐*S. aureus* antibody is conjugated to aminocellulose coated essential oil‐loaded zein nanocapsules (EO NCs) via a carboxyl‐to‐amine coupling reaction. Notably, up to twofold higher bactericidal efficacy was observed after treatment with Ab@EO NCs compared to the non‐targeted EO NCs under the same concentrations. Conjugation with specific antibodies enhances the interaction between EO NCs and the cellular surface, which increases the local essential oil concentration on the bacterial cell surface and therefore leads to rapid and efficient inactivation of *S. aureus*. The application of antibody‐conjugated nanomaterials in antibacterial treatment faces several requirements, such as high target specificity, affinity, efficient linkage, and stability. Nevertheless, the development of antibody‐conjugated nanomaterials for the purpose of clinical translation is highly attractive, as antibody‐based targeting ligands have overcome the problem of antibody immunogenicity in vivo.

### 4.2 Aptamer‐conjugated nanomaterials

Aptamers, often considered “chemical antibodies,” are short single‐stranded oligonucleotide selected by a process known as SELEX (systematic evolution of ligands by exponential enrichment) in vitro to target a range of specific ligands.^[127‐129^^]^ Aptamers usually consist of 15–60 nucleotides, which can form a specific secondary structure to recognize targets with high affinity and specificity. In the past few decades, aptamers have been intensively exploited as anticancer agents owing to their low cytotoxicity, easy preparation, and outstanding specific targeting ability.^[130‐132^^]^ Compared to antibodies, aptamers exhibit several favorable features^[133‐136^^]^: 1) aptamers can be prepared with great accuracy and reproducibility by chemical synthesis. Different from antibodies, aptamers can be easily functionalized with various functional groups via chemical reactions; 2) aptamers show higher thermal stability, resulting in recovering their native conformation and binding to target ligands after re‐annealing; 3) nucleic acids are usually not recognized as foreign agents by the human immune system, endowing aptamers with low immunogenicity; and 4) aptamers display a high specificity and affinity for some targets, such as small molecules and ions. Benefited from these features, aptamers markedly broaden their biological applications.

Aptamer‐conjugated NPs have been developed as attractive targeted therapeutic agents for treating bacterial infections. Antimicrobial peptides (AMP) have been designed and synthesized as alternative candidates for infection treatment by virtue of their outstanding bacterial inhibition efficacy in vitro. However, unsatisfactory in vivo performances, including instability, short half‐life, and weak penetration into mammalian cells, have restrained the clinical application of AMP.^[^ ^137^ ^]^ Lee et al. have reported aptamer‐conjugated AuNPs (AuNP‐Apt) for efficient delivery of AMP into mammalian living systems.^[^ ^138^ ^]^ Hexahistidine‐tag aptamers are conjugated to AuNPs loaded with C‐terminally His‐tagged AMP by simply mixing. The resulting AuNP‐Apt‐AMP is efficiently delivered into mammalian living systems. The ability to inhibit intracellular bacteria is further investigated in *S. Typhimurium* infected mice by intravenous injection and the results show that AuNP‐Apt‐AMP successfully eliminates *S. Typhimurium* colonization in organs and extend the survival of infected animals. Briefly, AuNP‐Apt‐AMP appear several advantages: 1) effective cellular entry; 2) increased stability of AMP by avoiding proteolysis; and 3) limited cytotoxicity to mammalian cells.

**FIGURE 8:**
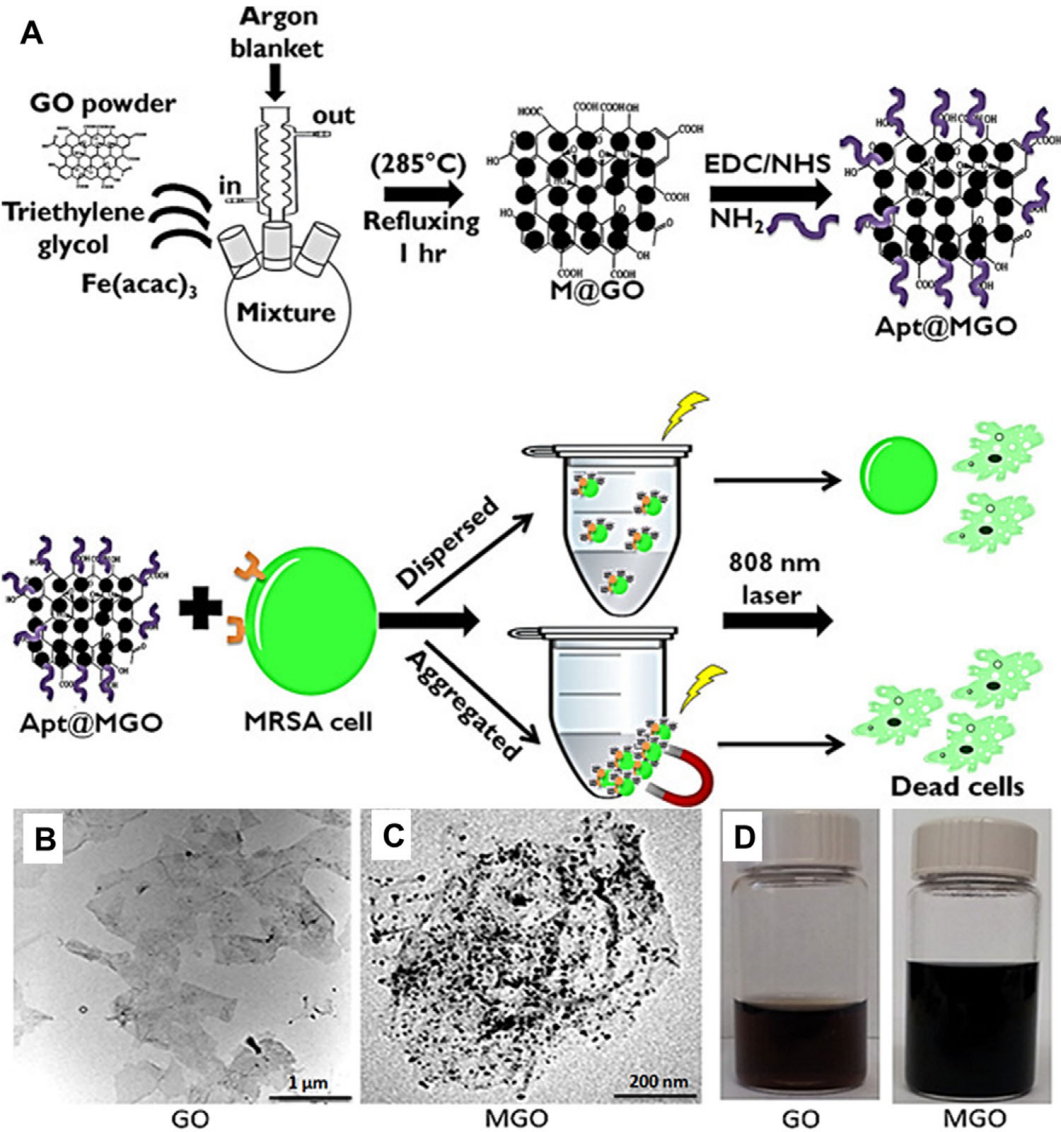
(A) Preparation of aptamer‐conjugated MGO and photothermal‐mediated inhibition of bacteria in both dispersed and aggregated forms in PBS under 808 nm NIR laser irradiation. TEM images of (B) bare GO and (C) MGO, respectively. (D) Digital photos of GO (left) and MGO (right) solutions, respectively. Reproduced with permission from.^[^ ^140^ ^]^ Copyright 2021, American Chemical Society

MRSA, as one of the most notorious human pathogens, can resist to different antibiotics. To enhance the therapeutic effects toward MRSA, aptamer‐conjugated gold nanorods (Apt@Au NRs) have been developed by Ocsoy et al.^[139^^]^ Apt@Au NRs can specifically bind to MRSA surface and effectively inactivate bacterial activity due to the high longitudinal absorption of NIR irradiation and strong photothermal conversion. The results also disclose that the binding strength and affinity to MRSA cells through a multivalent effect are increased with the number of aptamers conjugated to the surface of Au NPs. The same group has recently proposed an aptamer‐functionalized magnetic graphene oxide (Apt@MGO) for enhanced photothermal therapy against MRSA in both dispersed and aggregated states.^[^ ^140^ ^]^ Fe_3_O_4_ NPs are formed in situ on the surface of graphene oxide to prepare magnetic graphene oxide (MGO), which is used for collecting MRSA cells by an external magnet. MGO with aptamer conjugation is served as a photothermal agent to generate heat under NIR irradiation for MRSA deactivation (Figure 8). It has been observed that Apt@MGO separately kills 78% and 95% of MRSA in the dispersed and aggregated states after NIR laser irradiation for 200 seconds, highlighting that aptamer conjugation strengthens the selective and specific binding capability of MGO. Recently, Ozalp et al. have reported a new targeted delivery system of antibiotics to kill MRSA.^[^ ^141^ ^]^ To reduce antibiotic resistance development, targeted delivery systems have been used to retard the evolution of antibiotic resistance by restricting the dosage and administration frequency of antibiotics during treatment.^[142,^ ^142^, ^143^ ^]^ PLGA has been broadly used for drug delivery due to its biocompatibility and biodegradability.^[144‐146^^]^ In this context, the authors have prepared *S. aureus*‐specific aptamer‐conjugated PLGA NPs loaded with teicoplanin, which lead to 32‐ and 64‐fold decrements in minimum inhibitory concentration (MIC) against susceptible strains and clinical isolates of MRSA, respectively.

The existence of bacteria in aqueous environment raises concerns as a serious risk to human health. Traditional disinfectants, such as chlorine and ozone, usually form harmful by‐products.^[147‐149^^]^ By contrast, UV irradiation has been reported to potently disinfect bacteria with minimal formation of by‐products.^[150,^ ^150^, ^151^ ^]^ Kim et al. have constructed an *E. coli* surface‐specific aptamer cocktail (including three different aptamers that specifically target *E. coli*) with conjugated TiO_2_ particles (TiO_2_‐Apc) for targeted and increased inactivation of *E. coli*.^[^ ^152^ ^]^ Compared to single aptamer conjugated TiO_2_ (TiO_2_‐Aps) or non‐conjugated TiO_2_, TiO_2_‐Apc only require half of the time (30 mins) to inactivate ∼99% of *E. coli* (10^6^ CFU/mL) under UV irradiation. In addition to enhanced inhibition, aptamer cocktail‐conjugated TiO_2_ achieves increased bacterial selectivity than that of single‐type aptamer‐conjugated TiO_2_.

As an antibacterial agent, Ag is well known for its powerful antibacterial property, which is attributed to the release of Ag ions that present cytotoxicity to bacterial proteins, given that protein deactivation directly affects the generation of transmembrane ATP and ion transport across the cell membrane.^[153‐155^^]^ As nanoclusters (AgNCs) can attach to the bacterial cell wall and lead to cell death, their antibacterial ability is more potent against Gram‐negative bacteria.^[^ ^156^ ^]^ To widen the range of applications, Xu et al. have presented aptamer‐conjugated DNA‐templated AgNCs (AgNCs/Apt‐G) for accurate detection and effective elimination of *S. aureus*.^[^ ^157^ ^]^ As a “bridge,” an aptamer that specifically binds to *S. aureus* is connected to G‐rich sequences for enhancing fluorescent activity after attaching to AgNCs. The fluorescence intensity of N_x_‐AgNCs is greatly influenced by the number of complementary sequence bases. The results demonstrate that the strongest fluorescence intensity observed in N_4_‐AgNCs is increased by six‐times after aptamer conjugation. Compared to N_4_‐AgNCs treatment, N_4_‐AgNCs/Apt‐G display elevated antibacterial activity toward *S. aureus*.

**FIGURE 9:**
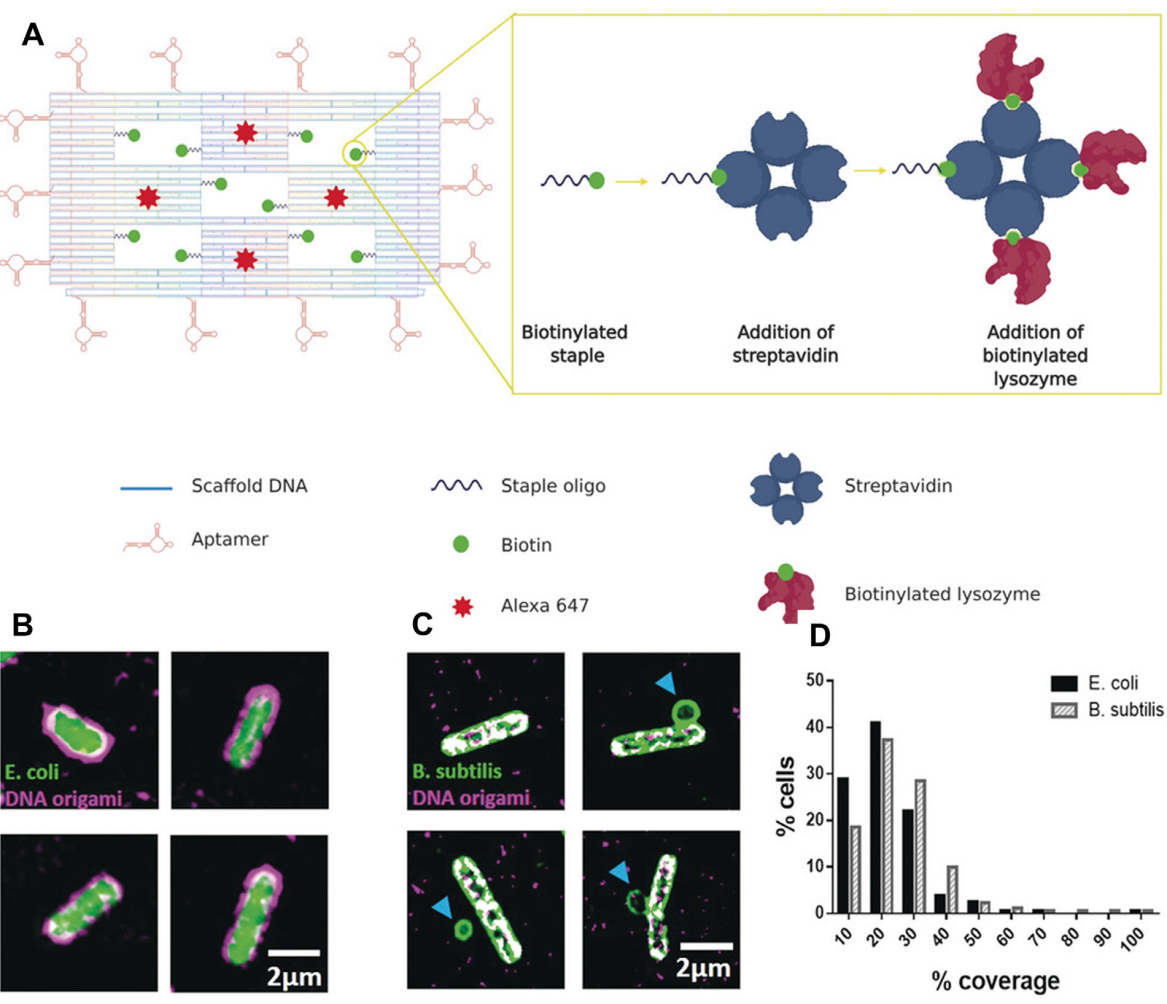
(A) Illustration of DNA origami nanostructure containing five “wells” carrying ten biotinylated staples for streptavidin attachment and fourteen aptamers conjugated with staples for driving the binding with bacteria. (B) Structured illumination microscopy (SIM) imaging of DNA origami (magenta) binding to *E. coli* (green). (C) SIM imaging of DNA origami (magenta) binding to *B. subtilis* (green). (D) Mean coverages for *E. coli* and *B. subtilis*, respectively. Reproduced with permission from.^[^ ^168^ ^]^ Copyright 2020, John Wiley & Sons

DNA origami structures, including two‐ or three‐dimensional nanostructures constructed by utilizing the base‐pairing property of DNA, have attracted plenty of attention for biomedical applications.^[158‐160^^]^ Owing to the characteristics of controllably designed geometries, excellent biocompatibility, and precise spatial addressability, DNA origami structures have been explored for drug delivery.^[161‐163^^]^ With the development of synthesis techniques, DNA origami structures, as a versatile nanoplatform, can deliver multiple payloads for targeted antibacterial delivery.^[164,^ ^164^, ^165^ ^]^ As reported, DNA origami structures have low immunogenicity and can remain intact over 48 h in vivo after specific design and modification.^[166,^ ^166^, ^167^ ^]^ Kaminski et al. have applied aptamer‐conjugated DNA origami nanostructures for the delivery of antibacterial peptide and lysozyme to specifically inactivate bacteria (Figure 9).^[^ ^168^ ^]^ DNA origami structures consisting of a frame with five “wells” for drug loading are conjugated with aptamers that can target *E. coli* and *Bacillus subtilis*. To ensure effective binding to target bacterial cells, fourteen aptamers are attached to each side of the nanostructure. With the loading of biotinylated lysozyme, this aptamer‐conjugated DNA origami shows significant growth inhibition of *E. coli* and *B. subtilis*, while no significant influence is observed on the viability of COS‐7 cells.

Recently, nanozymes have attracted increasing interest as a next generation of antibiotics due to high stability, broad spectrum antibacterial activity, limited occurrence of resistance, and low cost.^[169‐171^^]^ Compared to traditional antibiotics, nanozymes can be equipped with unique advantages of nanomaterials, such as reduced side effects and benign membrane permeability.^[172‐174^^]^ Different from natural enzymes, nanozymes profit from extra physicochemical properties, including size‐, composition‐, and shape‐modulating catalytic activities.^[175,^ ^175^, ^176^ ^]^ To date, a great number of antibacterial nanozymes, such as carbon‐based NPs, metal‐organic framework‐based compounds, transition metal peroxides/oxides, and metal‐based NPs, have been developed.^[177‐179^^]^ Yang et al. have developed an activatable aptamer‐conjugated nanozyme for targeted chemodynamic therapy of bacterial infection.^[^ ^180^ ^]^ Normally, chemodynamic therapy is based on the utilization of ROS to destroy cells by converting hydrogen peroxide (H_2_O_2_) into the highly toxic hydroxyl radical.^[181,^ ^181^, ^182^ ^]^ However, the peroxidase‐mimicking activity of most nanozymes is optimal in acidic solutions (pH 3–6), while most tissue environments are alkalescent.^[183,^ ^183^, ^184^ ^]^ To overcome this limitation, a nanocapsule (APGH) containing aptamer‐functionalized platinum nanozymes (Apt‐PtNZ), glucose oxidase (GOX), and hyaluronic acid (HA) is prepared for the antibacterial therapy of diabetic wounds. In vitro evaluation of antibacterial effects in PBS (pH 8.0) supplemented with 10 mM Glu (a simulated diabetic wound environment) shows that APGH can induce prominent morphology collapse of *S. aureus* through a dual aptamer‐targeting and glucose oxidation effect. An in vivo study in *S. aureus*‐infected diabetic mice further supports the ability of APGH to play antibacterial effect in alkalescent normal tissue environments. Despite the fact that various aptamers have been successfully conjugated to a variety of nanomaterials, the studies of antibacterial aptamers‐conjugated nanomaterials are currently in their infancy. It is expected that more antibacterial aptamer‐modified nanomaterials will become available for clinical trials in the near future.

### 4.3 Peptide‐conjugated nanomaterials

Peptides have been well‐known as targeting ligands for fighting infections because of their simple synthesis and high specificity.^[185,^ ^185^, ^186^ ^]^ Basically, the size of peptide ligands is between that of antibody and small molecular ligands. Peptides can simulate interactions among proteins and show a large binding area with receptors, resulting in remarkably higher specificity and binding affinity than molecular ligands. It is reported that antibacterial peptides display antibacterial functions by several strategies, such as delocalizing peripheral membrane proteins, inducing the permeability of cell membranes, and disrupting the organization of cell membrane.^[^ ^187^ ^]^ However, their potential applications are limited by hemolytic toxicity, low protease stability, and finite contact surface area.^[188,^ ^188^, ^189^ ^]^ The conjugation of peptides to NPs has been developed as an effective strategy to solve some of these difficulties.^[190‐192^^]^ Chen et al. have synthesized peptide‐conjugated reduced graphene oxide NPs (NRC03‐DA/nRGO) to enhance the stability of the NRC03 peptide for targeted treatment of bacterial infection.^[^ ^193^ ^]^ In addition to the increased stability of NRC03 peptide in physiological environments, conjugated NRC03‐DA/nRGO NPs exhibit ignorable cytotoxicity to BEAS‐2B cells after 24 h incubation, while free NRC03 peptide shows an apparent influence on cell viability under the same conditions. Furthermore, the antibacterial efficiency of these NPs toward Gram‐positive *S. aureus* is greatly enhanced after conjugation with the NRC03 peptide. To treat *S. aureus* infection, Tang et al. have reported an intracellular antibiotic delivery system (Gen@MSN@LU) as an active targeting agent for treating intracellular bacteria.^[^ ^194^ ^]^ Gen@MSN@LU is based on gentamicin‐loaded mesoporous silica NPs (Gen@MSNs) that are decorated with bacterial toxin‐responsive lipid bilayers and the targeting peptide UBI_29–41_. As a gate to control drug release, the lipid bilayers on the surface of Gen@MSN@LU are degraded by bacterial toxins specifically at the site of infection. The targeting and inhibition capabilities of Gen@MSN@LU toward *S. aureus* are confirmed both in vitro and in vivo.

Polymeric NPs represent an interesting approach for the delivery of antimicrobial drugs due to their controllable cargo release, facile surface functionalization, biodegradability, and low toxicity.^[^ ^195^ ^]^ Sullan et al. have reported a multifunctional and stimuli‐responsive polymeric antibacterial platform as an effective antibacterial strategy.^[^ ^196^ ^]^ This antibacterial platform (PdNP‐CWR11) is fabricated by decorating the surface of PDA NPs (PdNPs), a superior photothermal and biocompatible polymeric nanomaterial, with bacterial membrane‐targeting antimicrobial peptide CWR11. The combination of the membrane‐targeting ability of peptide CWR11 with the laser‐induced heating capability of PdNPs significantly triggered the cell death of *E. coli*, along with mitigating damage to healthy cells at the infection site. Lee et al. have reported a sort of self‐assembly micelles, which combines antimicrobial lipopeptide (DSPE‐PEG‐HnMc) and biodegradable amphiphilic polymers as new antibacterial agents.^[^ ^197^ ^]^ These micelles are prepared by the co‐self‐assembly of DSPE‐PEG‐HnMc and PLGA‐PEG, which form a hydrophobic PLGA and DSPE core as well as a hydrophilic PEG and HnMc shell. HnMc micelles display expected antibacterial activity against a wide spectrum of bacteria, demonstrating the great potential of antibacterial peptide‐based NPs for detection and treatment of bacterial infections. The translation potentiality of HnMc micelles as a targeted antibacterial agent is evaluated using a lung infection model, showing significantly improved antibacterial and anti‐inflammatory activities compared to free gentamicin. Although the antibacterial effectiveness of peptide‐conjugated nanomaterials has been proven in vitro, compromised antibacterial activity and decreased stability in vivo have hampered the clinical application. Furthermore, the cost and complexity of peptide synthesis are another challenge.

### 4.4 Other target‐specific nanomaterials

Polysaccharides, as an important type of natural biopolymers, have shown appealing properties benefitted from their stability, biodegradability, low toxicity, and diverse chemical modification feasibility.^[198,^ ^198^, ^199^ ^]^ Therefore, polysaccharides are considered as one type of the most promising building blocks for drug/gene delivery, bioimaging, and targeted treatment.^[^ ^200^ ^]^ The function and properties of polysaccharides depend on chain lengths, monosaccharide sequence, and charge. For instance, hyaluronic acid with negative charges has been widely developed as a targeting ligand, owing to its ability to bind with the CD44 receptor overexpressed on the surface of cancer cells. Moreover, hyaluronic acid has been reported to play an important role in anti‐inflammation. Pandey et al. have designed a hyaluronic acid‐conjugated self‐nano emulsifying drug delivery system (HA‐CIP‐SNEDDS) for improving the delivery of ciprofloxacin (CIP) toward *Salmonella* infection.^[^ ^201^ ^]^ To address the poor solubility and permeability of CIP, SNEDDS is used as a carrier, which successfully improves the permeability of HA‐CIP‐SNEDDS in goat intestinal mucus with the help of HA conjugation. Compared to free CIP, the permeability of HA‐CIP‐SNEDDS is increased fourfold after 4 h incubation, and around 80% of the CIP is released sustainably for 72 h. Additionally, Duan et al. have utilized HA‐conjugated metal organic framework (ZIF‐8) as a Trojan horse of vancomycin for treating MRSA infection.^[^ ^202^ ^]^ Competitive residency experiments demonstrate that HA‐conjugated ZIF‐8 can be endocytosed by macrophages via specifically binding to CD44 for targeted clearance of bacteria in vitro. Moreover, in vivo antibacterial results indicate that HA‐conjugated ZIF‐8 has more effective inhibition of MRSA than the same dose of vancomycin.

Small molecular ligands have been an important medium by virtue of their simple structure and definite interactions with receptors. For instance, vancomycin, as a well‐recognized small molecular targeting ligand, has been conjugated to the surfaces of AuNPs,^[^ ^203^ ^]^ iron oxides,^[^ ^204^ ^]^ dendrimers,^[^ ^205^ ^]^ and microporous silica NPs^[^ ^206^ ^]^ for specific binding with Gram‐positive bacteria. Li et al. have designed pH/lipase‐sensitive micellar carriers encapsulated with ciprofloxacin (Van‐hyd‐PECL/CiP), which are prepared by self‐assembly of amphiphilic poly(ethylene glycol)‐poly(ε‐caprolactone) (PECL) copolymers conjugated with vancomycin (VAN).^[^ ^207^ ^]^ The improved blood circulation time and selective recognition are ascribed to the presence of PEG shell and conjugated VAN targeting ligand. In vivo results imply that the survival of *P. aeruginosa*‐infected mice after treatment with Van‐hyd‐PECL/Cip is largely prolonged than those of mice treated with either free drugs or micelles without VAN conjugation. This work provides a feasible strategy to use VAN as a small molecular ligand for targeted therapy of bacterial infections. Folic acid (FA), also known as vitamin B9, has been employed as a specific targeting ligand against bacteria.^[^ ^208^, ^209^ ^]^ Zhang et al. have prepared a multifunctional nanocarrier (Oxi‐αCD NPs) with a DSPE‐PEG‐FA coating for targeted delivery of moxifloxacin (MXF) to pulmonary *P. aeruginosa*.^[^ ^210^ ^]^ Higher cell internalization of MXF/FA‐Oxi‐αCD NPs by macrophages is observed as the attachment of FA on NP surface renders stronger adhesion to folate receptors presenting on macrophages. Meanwhile, the biodistribution analysis exhibits that MXF/FA‐Oxi‐αCD NPs mainly accumulate in the infected tissue, owing to the overexpression of folate receptors on activated macrophages. As a result, MXF/FA‐Oxi‐αCD NPs prolong the survival time of pulmonary‐infected mice.

Moreover, magnetic targeting nanomaterials have recently ushered into an increasingly attractive research area. Superparamagnetic iron oxide NPs, consisting of iron oxide cores, can become magnetized with an external magnetic field. In this way, superparamagnetic iron oxide NPs are endowed with the capability to deliver the coated or conjugated drugs to the target tissue under an external magnetic field guidance and can be inactivated after removing the magnetic field. Recently, magnetic drug nano‐delivery systems, such as magnetodendrimers, magnetoliposomes, and magnetic nanocomposite, have been introduced to reduce the menace of resistant bacteria.^[211,^ ^211^, ^212^ ^]^ For example, Markiewicz et al. have used a polymer/gold/magnetic‐NPs nanocomposite to inhibit the metabolic activity of *P. aeruginosa* and prevent the formation of biofilm.^[^ ^213^ ^]^ Sahoo et al. have investigated a magnetic nanocomposite, Congo Red dye loaded Fe_3_O_4_‐*N*‐[3‐(trimethoxysilyl)‐ propyl]‐ethylenediamine‐Tryptophan (FTT), for bacterial inhibition.^[^ ^214^ ^]^ For specific site targeting, the antibacterial activity of FTT nanocomposite was examined against Gram‐negative (*E. coli*) and Gram‐positive (*B. subtilis*) bacterial strain, displaying positive antibacterial responses. However, to develop high‐quality antibacterial targeting strategies remains one of the most important challenges for targeted antibacterial therapy based on nanomaterials.

## 5 CONCLUSION AND OUTLOOK

Antibiotics have saved a great number of lives from infectious diseases every year. At the same time, the wide use of antibiotics causes an alarming spread of bacterial resistance to antibiotics, which is currently a global health emergency. It is clear that the introduction of more powerful antibiotics would not be the eventual solution to overcome antibiotic resistance, which in turn may accelerate the development of greater and more severe resistance. Given the advances in nanotechnology and material science, various nanomaterials have been developed as an alternative to against antibiotic resistant bacteria, due to their high surface‐to‐volume ratio and unique physicochemical properties. Despite the fact that antibacterial mechanisms are not yet well understood, it is clarified that nanomaterials not only are equipped with intrinsic antibacterial activity but also can be used as vehicles for the efficient delivery of antibacterial agents. Targeting bacteria is one of the most widely utilized approaches, which can increase the local concentration of antimicrobial agents around bacteria cells and result in enhanced inhibition effects and reduced side effects. In terms of passive targeting, nanomaterials depend on sufficient blood circulation time to accumulate in target tissues, attributing to their specific physicochemical property as well as unique responsive nature in target sites. To further enhance drug delivery to infected lesions, targeting ligands are conjugated to the surface of nanomaterials to recognize specific receptors expressed on target cells. Both passive and active strategies that target infection sites can increase delivery efficiency, which can reduce side effects and enhance antibacterial ability simultaneously.

Despite these progresses, clinical translation of antibacterial nanomaterials is in preliminary stage. A few antibacterial AgNPs or nanocarriers for antibiotic delivery have been undergoing clinical testing.^[^ ^215^ ^]^ For example, Pulmaquin, a liposomal nanoformulation designed for controlled delivery of ciprofloxacin, is in phase III clinical trials.^[^ ^216^ ^]^ However, many hurdles and challenges hamper the translation of antibacterial nanomaterials into clinical applications. Firstly, the antibacterial mechanisms of nanomaterials are bottlenecks to deeply elucidate effectiveness at the molecular level. The development of molecular dynamics simulations provides opportunities to fully elucidate the structure‐activity relationship and antibacterial mechanisms of nanomaterials. Second, the antibacterial capability of nanomaterials is inadequate to completely meet the requirements of clinical applications. Not only inherent physicochemical properties but also extrinsic factors affect the antibacterial activity of nanomaterials. Therefore, optimal nanomaterials with high antibacterial activity in biological systems are highly desirable. Last, most nanomaterials present excellent antibacterial inhibition in vitro but lack significant progress in clinical translations due to the complexity of biological systems. Given the complexity of biological environments, more investigations regarding in vivo biodistribution, metabolic pathways, and degradation of nanomaterials have become critical for future application. While nanomaterials hold great promise in fighting bacterial infection, knowledge regarding the pharmacokinetic profiles and safety issues of nanomaterials applied to the human body is extremely limited. With increasing the understanding of interdisciplinary in nanotechnology, microbiology, pharmacology, immunology, and toxicology, targeted antibacterial therapy would present a major leap forward and be able to confront the threat of antibiotic‐resistant bacteria.

## CONFLICT OF INTEREST

The authors declare no conflict of interest.

## References

[1] WHO , https://www.who.int/news‐room/fact‐sheets/detail/the‐top‐10‐causes‐of‐death (Accessed: December 2020).

[2] N. Komerik , A. J. MacRobert , J. Environ. Pathol. Toxicol. Oncol. 2006, 25, 487. 16566737 10.1615/jenvironpatholtoxicoloncol.v25.i1-2.310

[3] S. Afrasiabi , M. Pourhajibagher , R. Raoofian , M. Tabarzad , A. Bahador , J. Biomed. Sci. 2020, 27, 6. 31900238 10.1186/s12929-019-0611-0 PMC6941257

[4] S. B. Levy , B. Marshall , Nat. Med. 2004, 10, S122. 15577930 10.1038/nm1145

[5] Y. Wang , Y. Yang , Y. Shi , H. Song , C. Yu , Adv. Mater. 2020, 32, 1904106. 10.1002/adma.201904106 31799752

[6] P. Maria , G. Tanzi , Jinri Yaoxue 2020, 26, 14.

[7] X. Zhen , C. S. Lundborg , X. Sun , X. Hu , H. Dong , Antimicrob. Resist. Infect. Control. 2019, 8, 137. 31417673 10.1186/s13756-019-0590-7 PMC6692939

[8] B. D. Brooks , A. E. Brooks , Adv. Drug Delivery Rev. 2014, 78, 14. 10.1016/j.addr.2014.10.027 25450262

[9] K. H. Luepke , K. J. Suda , H. Boucher , R. L. Russo , M. W. Bonney , T. D. Hunt , J. F. Mohr III , Pharmacotherapy 2017, 37, 71. 27859453 10.1002/phar.1868

[10] Y. Sun , R. Zhou , L. Meng , Y. Zhang , D. Zhao , X. Cai , Curr. Stem Cell Res. Ther. 2020, 15, 66. 10.2174/1574888X15666200521084417 32436832

[11] M. F. Chellat , L. Raguž , R. Riedl , Angew. Chem. Int. Ed. 2016, 55, 6600. 10.1002/anie.201506818 PMC5071768 27000559

[12] R. C. Allen , R. Popat , S. P. Diggle , S. P. Brown , Nat. Rev. Microbiol. 2014, 12, 300. 24625893 10.1038/nrmicro3232

[13] L. L. Silver , Nat. Rev. Drug Discovery. 2007, 6, 41. 17159922 10.1038/nrd2202

[14] E. K. Saeki , R. K. T. Kobayashi , G. Nakazato , Microb. Pathog. 2020, 142, 104068. 32061914 10.1016/j.micpath.2020.104068

[15] S. Gunaratnam , M. Millette , L. V. McFarland , H. L. DuPont , M. Lacroix , Microb. Pathog. 2021, 153, 104798. 33609647 10.1016/j.micpath.2021.104798

[16] European Observatory on Health Systems, Policies , M. Renwick , E. Mossialos , Eurohealth 2020, 26, 8. 28806044

[17] A. Frei , J. Zuegg , A. G. Elliott , M. Baker , S. Braese , C. Brown , F. Chen , C. G. Dowson , G. Dujardin , N. Jung , A. P. King , A. M. Mansour , M. Massi , J. Moat , H. A. Mohamed , A. K. Renfrew , P. J. Rutledge , P. J. Sadler , M. H. Todd , C. E. Willans , J. J. Wilson , M. A. Cooper , M. A. T. Blaskovich , Chem. Sci. 2020, 11, 2627. 32206266 10.1039/c9sc06460e PMC7069370

[18] J. Lakshmaiah Narayana , B. Mishra , T. Lushnikova , Q. Wu , Y. S. Chhonker , Y. Zhang , D. Zarena , E. S. Salnikov , X. Dang , F. Wang , C. Murphy , K. W. Foster , S. Gorantla , B. Bechinger , D. J. Murry , G. Wang , Proc. Natl. Acad. Sci. U. S. A. 2020, 117, 19446. 32723829 10.1073/pnas.2005540117 PMC7431008

[19] M. Miethke , M. Pieroni , T. Weber , M. Brönstrup , P. Hammann , L. Halby , P. B. Arimondo , P. Glaser , B. Aigle , H. B. Bode , R. Moreira , Y. Li , A. Luzhetskyy , M. H. Medema , J.‐L. Pernodet , M. Stadler , J. R. Tormo , O. Genilloud , A. W. Truman , K. J. Weissman , E. Takano , S. Sabatini , E. Stegmann , H. Brötz‐Oesterhelt , W. Wohlleben , M. Seemann , M. Empting , A. K. H. Hirsch , B. Loretz , C.‐M. Lehr , et al. , Nat. Rev. Chem. 2021, 5, 726. 10.1038/s41570-021-00313-1 PMC8374425 34426795

[20] I. Yacoby , H. Bar , I. Benhar , Antimicrob. Agents Chemother. 2007, 51, 2156. 17404004 10.1128/AAC.00163-07 PMC1891362

[21] N. Bertrand , J. Wu , X. Xu , N. Kamaly , O. C. Farokhzad , Adv. Drug Delivery Rev. 2014, 66, 2. 10.1016/j.addr.2013.11.009 PMC4219254 24270007

[22] A. D. Friedman , S. E. Claypool , R. Liu , Curr. Pharm. Des. 2013, 19, 6315. 23470005 10.2174/13816128113199990375 PMC4016770

[23] C. Deusenbery , Y. Wang , A. Shukla , ACS Infect. Dis. 2021, 7, 695. 33733747 10.1021/acsinfecdis.0c00890

[24] P. Jelinkova , A. Mazumdar , V. P. Sur , S. Kociova , K. Dolezelikova , A. M. J. Jimenez , Z. Koudelkova , P. K. Mishra , K. Smerkova , Z. Heger , M. Vaculovicova , A. Moulick , V. Adam , J. Controlled Release 2019, 307, 166. 10.1016/j.jconrel.2019.06.013 31226356

[25] L. Wang , C. Hu , L. Shao , Int. J. Nanomed. 2017, 12, 1227. 10.2147/IJN.S121956 PMC5317269 28243086

[26] C. Li , R. Ye , J. Bouckaert , A. Zurutuza , D. Drider , T. Dumych , S. Paryzhak , V. Vovk , R. O. Bilyy , S. Melinte , M. Li , R. Boukherroub , S. Szunerits , ACS Appl. Mater. Interfaces 2017, 9, 36665. 28956593 10.1021/acsami.7b12949

[27] W. Gao , L. Zhang , Nat. Rev. Microbiol. 2021, 19, 5. 33024312 10.1038/s41579-020-00469-5 PMC7538279

[28] A. Gupta , S. Mumtaz , C.‐H. Li , I. Hussain , V. M. Rotello , Chem. Soc. Rev. 2019, 48, 415. 30462112 10.1039/c7cs00748e PMC6340759

[29] Y. Xing , W. Li , Q. Wang , X. Li , Q. Xu , X. Guo , X. Bi , X. Liu , Y. Shui , H. Lin , H. Yang , Molecules 2019, 24, 1695. 31052263 10.3390/molecules24091695 PMC6539459

[30] N. Saadi , K. Alotaibi , L. Hassan , Q. Smith , F. Watanabe , A. Khan , T. Karabacak , Nanotechnology 2021, 32, 325103. 10.1088/1361-6528/abfd59 33930890

[31] J. C. Medina , V. I. Garcia‐Perez , R. Zanella , Mater. Today Commun. 2021, 26, 102182.

[32] A. Verma , S. Shivalkar , M. P. Sk , S. K. Samanta , A. K. Sahoo , Nanotechnology 2020, 31, 405704. 32498056 10.1088/1361-6528/ab996f

[33] H. Li , Y. Zou , J. Jiang , Dalton Trans. 2020, 49, 9274. 32572419 10.1039/d0dt01816c

[34] Y. Xiao , T. Zhang , Y. Li , C. Liu , S. Yang , L. Zeng , Microb. Drug Resist. 2020, 28, 7. 10.1089/mdr.2020.0511 34357802

[35] C. I. Colino , J. M. Lanao , C. Gutierrez‐Millan , Mater. Sci. Eng. C 2021, 121, 111843. 10.1016/j.msec.2020.111843 33579480

[36] Y. Wang , Y. Yang , Y. Shi , H. Song , C. Yu , Adv. Mater. 2020, 32, 1904106. 10.1002/adma.201904106 31799752

[37] W. Fan , H. Han , Y. Chen , X. Zhang , Y. Gao , S. Li , Q. Jin , J. Ji , K. Yao , Drug Delivery Transl. Rev. 2021, 11, 1352. 10.1007/s13346-021-00966-x 33840082

[38] A. Ivanova , K. Ivanova , J. Hoyo , T. Heinze , S. Sanchez‐Gomez , T. Tzanov , ACS Appl. Mater. Interfaces 2018, 10, 3314. 29313670 10.1021/acsami.7b16508

[39] X. Lu , X. Feng , J. R. Werber , C. Chu , I. Zucker , J.‐H. Kim , C. O. Osuji , M. Elimelech , Proc. Natl. Acad. Sci. U. S. A. 2017, 114, E9793. 29078354 10.1073/pnas.1710996114 PMC5699062

[40] P. Kumar , P. Huo , R. Zhang , B. Liu , Nanomaterials 2019, 9, 737. 31086043 10.3390/nano9050737 PMC6567318

[41] E. P. Ivanova , J. Hasan , H. K. Webb , V. K. Truong , G. S. Watson , J. A. Watson , V. A. Baulin , S. Pogodin , J. Y. Wang , M. J. Tobin , C. Löbbe , R. J. Crawford , Small 2012, 8, 2489. 22674670 10.1002/smll.201200528

[42] Y. Li , H. Yuan , A. von dem Bussche , M. Creighton , R. H. Hurt , A. B. Kane , H. Gao , Proc. Natl. Acad. Sci. U. S. A. 2013, 110, 12295. 23840061 10.1073/pnas.1222276110 PMC3725082

[43] Q. Borjihan , A. Dong , Biomater. Sci. 2020, 8, 6867. 32756731 10.1039/d0bm00788a

[44] H. Ji , H. Sun , X. Qu , Adv. Drug Delivery Rev. 2016, 105, 176. 10.1016/j.addr.2016.04.009 27129441

[45] B. Ren , K. Li , Z. Liu , G. Liu , H. Wang , J. Mater. Chem. B 2020, 8, 10754. 33155608 10.1039/d0tb02272a

[46] A. M. Rice , Y. Long , S. B. King , Biomolecules 2021, 11, 267. 33673069 10.3390/biom11020267 PMC7918234

[47] N. Kwon , D. Kim , K. M. K. Swamy , J. Yoon , Coord. Chem. Rev. 2021, 427, 213581.

[48] S. Liu , K. Lv , Z. Chen , C. Li , T. Chen , D. Ma , Biomater. Sci. 2021, 9, 6486. 34582524 10.1039/d1bm01077h

[49] W. R. Rolim , J. C. Pieretti , D. L. S. Renó , B. A. Lima , M. H. M. Nascimento , F. N. Ambrosio , C. B. Lombello , M. Brocchi , A. C. S. de Souza , A. B. Seabra , ACS Appl. Mater. Interfaces 2019, 11, 6589. 30653288 10.1021/acsami.8b19021

[50] M. Li , W. Qiu , Q. Wang , N. Li , X. Wang , J. Yu , X. Li , F. Li , D. Wu , Composites Part B 2022, 229, 109484.

[51] M. T. Pelegrino , J. C. Pieretti , G. Nakazato , M. C. Gonçalves , J. C. Moreira , A. B. Seabra , Nitric Oxide 2021, 106, 24. 33098968 10.1016/j.niox.2020.10.003

[52] J. W. Xu , K. Yao , Z. K. Xu , Nanoscale 2019, 11, 8680. 31012895 10.1039/c9nr01833f

[53] Q. Xin , H. Shah , A. Nawaz , W. Xie , M. Z. Akram , A. Batool , L. Tian , S. U. Jan , R. Boddula , B. Guo , Q. Liu , J. R. Gong , Adv. Mater. 2019, 31, 1804838. 10.1002/adma.201804838 30379355

[54] R. Zhang , J. Yu , K. Ma , Y. Ma , Z. Wang , ACS Appl. Mater. Interfaces 2020, 3, 7168. 10.1021/acsabm.0c00979 35019375

[55] S. Zhang , Q. Lu , F. Wang , Z. Xiao , L. He , D. He , L. Deng , ACS Appl. Mater. Interfaces 2021, 13, 37535. 34324300 10.1021/acsami.1c10600

[56] İ. Aksoy , H. Küçükkeçeci , F. Sevgi , Ö. Metin , I. Hatay Patir , ACS Appl. Mater. Interfaces 2020, 12, 26822. 32427479 10.1021/acsami.0c02524

[57] Z. M. Marković , S. P. Jovanović , P. Z. Mašković , M. Danko , M. Mičušík , V. B. Pavlović , D. D. Milivojević , A. Kleinová , Z. Špitalský , B. M. Todorović Marković , RSC Adv. 2018, 8, 31337. 35548242 10.1039/c8ra04664f PMC9085601

[58] H. Lv , Y. Zhang , P. Chen , J. Xue , X. Jia , J. Chen , Langmuir 2020, 36, 4025. 32216361 10.1021/acs.langmuir.0c00292

[59] M. Kováčová , E. Špitalská , Z. Markovic , Z. Špitálský , Part. Part. Syst. Charact. 2020, 37, 1900348.

[60] W. Huang , F. Tao , F. Li , M. Mortimer , L.‐H. Guo , NanoImpact 2020, 20, 100268.

[61] L. Marín‐Caba , G. Bodelón , Y. Negrín‐Montecelo , M. A. Correa‐Duarte , Adv. Funct. Mater. 2021, 31, 2105807.

[62] M. Gholipourmalekabadi , M. Mobaraki , M. Ghaffari , A. Zarebkohan , V. Fallah Omrani , A. Urbanska , A. Seifalian , Curr. Pharm. Des. 2017, 23, 2918. 28425863 10.2174/1381612823666170419105413

[63] Y. C. Yeh , T. H. Huang , S. C. Yang , C. C. Chen , J. Y. Fang , Front. Chem. 2020, 8, 286. 32391321 10.3389/fchem.2020.00286 PMC7193053

[64] A. S. Joshi , P. Singh , I. Mijakovic , Int. J. Mol. Sci. 2020, 21, 7658. 33081366 10.3390/ijms21207658 PMC7589962

[65] S. Roy , A. Mondal , V. Yadav , A. Sarkar , R. Banerjee , P. Sanpui , A. Jaiswal , ACS Appl. Bio Mater. 2019, 2, 2738. 10.1021/acsabm.9b00124 35030809

[66] H. N. Abdelhamid , H.‐F. Wu , TrAC, Trends Anal. Chem. 2015, 65, 30.

[67] Y. Cao , S. Moniri Javadhesari , S. Mohammadnejad , E. Khodadustan , A. Raise , M. R. Akbarpour , Ceram. Int. 2021, 47, 25729.

[68] A. N. Coman , A. Mare , C. Tanase , E. Bud , A. Rusu , Metals 2021, 11, 92.

[69] D.‐N. Phan , N. Dorjjugder , Y. Saito , M. Q. Khan , A. Ullah , X. Bie , G. Taguchi , I.‐S. Kim , Mater. Today Commun. 2020, 25, 101377.

[70] M. Hashmi , S. Ullah , I. S. Kim , Mater. Today Commun. 2020, 24, 101161.

[71] W. Yu , X. Li , J. He , Y. Chen , L. Qi , P. Yuan , K. Ou , F. Liu , Y. Zhou , X. Qin , J. Colloid Interface Sci. 2021, 584, 164. 33069016 10.1016/j.jcis.2020.09.092

[72] S.‐L. Bee , Y. Bustami , A. Ul‐Hamid , K. Lim , Z. A. Abdul Hamid , J. Mater. Sci. Mater. Med. 2021, 32, 106. 34426879 10.1007/s10856-021-06590-y PMC8382650

[73] W. Gao , S. Thamphiwatana , P. Angsantikul , L. Zhang , Wiley Interdiscip. Rev. Nanomed. Nanobiotechnol. 2014, 6, 532. 25044325 10.1002/wnan.1282 PMC4197093

[74] A. J. Huh , Y. J. Kwon , J. Controlled Release 2011, 156, 128. 10.1016/j.jconrel.2011.07.002 21763369

[75] O. A. Madkhali , S. Sivagurunathan Moni , M. H. Sultan , H. A. Bukhary , M. Ghazwani , N. A. Alhakamy , A. M. Meraya , S. Alshahrani , S. S. Alqahtani , M. A. Bakkari , M. I. Alam , M. E. Elmobark , Sci. Rep. 2021, 11, 9914. 33972626 10.1038/s41598-021-89330-0 PMC8110975

[76] S. O. Meroueh , K. Z. Bencze , D. Hesek , M. Lee , J. F. Fisher , T. L. Stemmler , S. Mobashery , Proc. Natl. Acad. Sci. U. S. A. 2006, 103, 4404. 16537437 10.1073/pnas.0510182103 PMC1450184

[77] J. Y. Cheon , S. J. Kim , Y. H. Rhee , O. H. Kwon , W. H. Park , Int. J. Nanomed. 2019, 14, 2773. 10.2147/IJN.S196472 PMC6499446 31118610

[78] A. Naskar , K. S. Kim , Pharmaceutics 2020, 12, 86.31972960

[79] A. Naskar , S. Lee , K.‐s. Kim , Pharmaceutics 2021, 13, 52. 33401709 10.3390/pharmaceutics13010052 PMC7823710

[80] O. T. Fanoro , S. Parani , R. Maluleke , T. C. Lebepe , R. J. Varghese , N. Mgedle , V. Mavumengwana , O. S. Oluwafemi , Antibiotics 2021, 10, 1275. 34827214 10.3390/antibiotics10111275 PMC8614812

[81] J. Suresh , S. I. Hong , Adv. Mater. 2014, 952, 137.

[82] S. F. Hashemi , N. Tasharrofi , M. M. Saber , J. Mol. Struct. 2020, 1208, 127889.

[83] O. Polivanova , M. Cherednichenko , E. Kalashnikova , R. Kirakosyan , AIMS Agric. Food 2021, 6, 631.

[84] S. Ahmadi , M. Fazilati , H. Nazem , S. M. Mousavi , BioMed. Res. Int. 2021, 2021, 8822645. 33542927 10.1155/2021/8822645 PMC7840253

[85] M. Ramzan , R. M. Obodo , S. Mukhtar , S. Z. Ilyas , F. Aziz , N. Thovhogi , Mater. Today 2021, 36, 576.

[86] A. Fatiqin , H. Amrulloh , W. Simanjuntak , Bull. Chem. Soc. Ethiop. 2021, 35, 161.

[87] A. Saravanan , M. Maruthapandi , P. Das , J. H. T. Luong , A. Gedanken , Nanomaterials 2021, 11, 369. 33540607 10.3390/nano11020369 PMC7912860

[88] M. Chamundeeswari , J. Jeslin , M. L. Verma , Environ. Chem. Lett. 2019, 17, 849.

[89] E. A. Azzopardi , E. L. Ferguson , D. W. Thomas , J. Antimicrob. Chemother. 2013, 68, 257. 23054997 10.1093/jac/dks379

[90] A. Vassallo , M. F. Silletti , I. Faraone , L. Milella , J. Nanomater. 2020, 2020, 6905631.

[91] C. C. Liao , H. P. Yu , S. C. Yang , A. Alalaiwe , Y. S. Dai , F. C. Liu , J. Y. Fang , J. Nanobiotechnol. 2021, 19, 48. 10.1186/s12951-021-00789-5 PMC7885212 33588861

[92] Y. Jaglal , N. Osman , C. A. Omolo , C. Mocktar , N. Devnarain , T. Govender , J. Drug Delivery Sci. Technol. 2021, 64, 102607.

[93] Q. Cai , Y. Fei , H. W. An , X. X. Zhao , Y. Ma , Y. Cong , L. Hu , L. L. Li , H. Wang , ACS Appl. Mater. Interfaces 2018, 10, 9197. 29443494 10.1021/acsami.7b19056

[94] Y. Fan , X. D. Li , P. P. He , X. X. Hu , K. Zhang , J. Q. Fan , P. P. Yang , H. Y. Zheng , W. Tian , Z. M. Chen , L. Ji , H. Wang , L. Wang , Sci. Adv. 2020, 6, eaaz4767. 32494712 10.1126/sciadv.aaz4767 PMC7209993

[95] H.‐W. An , Y. Fei , T.‐D. Yan , C.‐Q. Lu , M.‐D. Wang , T. Ma , B.‐Y. Zhao , J.‐M. Nie , H.‐R. Tseng , L.‐L. Li , H. Wang , Adv. Ther. 2020, 3, 1900217.

[96] Y. Liang , H. Zhu , L. Wang , H. He , S. Wang , Carbohydr. Polym. 2020, 249, 116876. 32933696 10.1016/j.carbpol.2020.116876

[97] H. D. Summers , P. Rees , M. D. Holton , M. Rowan Brown , S. C. Chappell , P. J. Smith , R. J. Errington , Nat. Nanotechnol. 2011, 6, 170. 21258333 10.1038/nnano.2010.277

[98] M. Gholipourmalekabadi , M. Mobaraki , M. Ghaffari , A. Zarebkohan , V. F. Omrani , A. M. Urbanska , A. Seifalian , Curr. Pharm. Des. 2017, 23, 2918. 28425863 10.2174/1381612823666170419105413

[99] J. D. Byrne , T. Betancourt , L. Brannon‐Peppas , Adv. Drug Delivery Rev. 2008, 60, 1615. 10.1016/j.addr.2008.08.005 18840489

[100] C. Wang , Y. Wang , L. Zhang , R. J. Miron , J. Liang , M. Shi , W. Mo , S. Zheng , Y. Zhao , Y. Zhang , Adv. Mater. 2018, 30, 1804023. 10.1002/adma.201804023 30285289

[101] T. Kobayashi , T. Ishida , Y. Okada , S. Ise , H. Harashima , H. Kiwada , Int. J. Pharm. 2007, 329, 94. 16997518 10.1016/j.ijpharm.2006.08.039

[102] C. Bebbington , G. Yarranton , Curr. Opin. Biotechnol. 2008, 19, 613. 19000762 10.1016/j.copbio.2008.10.002

[103] D. M. Francis , M. P. Manspeaker , P. A. Archer , L. F. Sestito , A. J. Heiler , A. Schudel , S. N. Thomas , Biomaterials 2021, 279, 121184. 34678650 10.1016/j.biomaterials.2021.121184 PMC8639654

[104] M. Zu , Y. Ma , B. Cannup , D. Xie , Y. Jung , J. Zhang , C. Yang , F. Gao , D. Merlin , B. Xiao , Adv. Drug Delivery Rev. 2021, 176, 113887. 10.1016/j.addr.2021.113887 34314785

[105] A. S. Schmid , D. Neri , Nat. Rev. Rheumatol. 2019, 15, 197. 30814691 10.1038/s41584-019-0188-8

[106] P. K. Jain , X. Huang , I. H. El‐Sayed , M. A. El‐Sayed , Acc. Chem. Res. 2008, 41, 1578. 18447366 10.1021/ar7002804

[107] G. V. Vimbela , S. M. Ngo , C. Fraze , L. Yang , D. A. Stout , Int. J. Nanomed. 2017, 12, 3941. 10.2147/IJN.S134526 PMC5449158 28579779

[108] R. S. Norman , J. W. Stone , A. Gole , C. J. Murphy , T. L. Sabo‐Attwood , Nano Lett. 2008, 8, 302. 18062714 10.1021/nl0727056

[109] S. Wang , A. K. Singh , D. Senapati , A. Neely , H. Yu , P. C. Ray , Chemistry 2010, 16, 5600. 20397252 10.1002/chem.201000176

[110] J. Chen , S. M. Andler , J. M. Goddard , S. R. Nugen , V. M. Rotello , Chem. Soc. Rev. 2017, 46, 1272. 27942636 10.1039/c6cs00313c PMC5339056

[111] V. P. Zharov , K. E. Mercer , E. N. Galitovskaya , M. S. Smeltzer , Biophys. J. 2006, 90, 619. 16239330 10.1529/biophysj.105.061895 PMC1367066

[112] D. G. Meeker , S. V. Jenkins , E. K. Miller , K. E. Beenken , A. J. Loughran , A. Powless , T. J. Muldoon , E. I. Galanzha , V. P. Zharov , M. S. Smeltzer , J. Chen , ACS Infect. Dis. 2016, 2, 241. 27441208 10.1021/acsinfecdis.5b00117 PMC4945994

[113] D. G. Meeker , T. Wang , W. N. Harrington , V. P. Zharov , S. A. Johnson , S. V. Jenkins , S. E. Oyibo , C. M. Walker , W. B. Mills , M. E. Shirtliff , K. E. Beenken , J. Chen , M. S. Smeltzer , Int. J. Hyperthermia 2018, 34, 209. 29025325 10.1080/02656736.2017.1392047 PMC6095133

[114] T. Mahmoudi , M. Pourhassan‐Moghaddam , B. Shirdel , B. Baradaran , E. Morales‐Narváez , H. Golmohammadi , J. Mater. Chem. B 2021, 9, 5414. 34143173 10.1039/d1tb00571e

[115] N. Levi‐Polyachenko , C. Young , C. McNeill , A. Braden , L. Argenta , S. Reid , Int. J. Hyperthermia 2014, 30, 490. 25354678 10.3109/02656736.2014.966790 PMC11371122

[116] Y.‐W. Wang , Y.‐Y. Fu , L.‐J. Wu , J. Li , H.‐H. Yang , G.‐N. Chen , J. Mater. Chem. B 2013, 1, 2496. 32261050 10.1039/c3tb20144a

[117] T. J. Ondera , A. T. Hamme Ii , J. Mater. Chem. B 2014, 2, 7534. 25414794 10.1039/C4TB01195C PMC4234150

[118] N. Beyth , Y. Houri‐Haddad , A. Domb , W. Khan , R. Hazan , J. Evidence‐Based Complementary Altern. Med. 2015, 2015, 246012. 10.1155/2015/246012 PMC4378595 25861355

[119] S. M. Dizaj , F. Lotfipour , M. Barzegar‐Jalali , M. H. Zarrintan , K. Adibkia , Mater. Sci. Eng. C 2014, 44, 278. 10.1016/j.msec.2014.08.031 25280707

[120] Y.‐N. Gao , Y. Wang , T.‐N. Yue , Y.‐X. Weng , M. Wang , J. Colloid Interface Sci. 2021, 582, 112. 32814219 10.1016/j.jcis.2020.08.037

[121] M.‐M. Zhu , Y. Fang , Y.‐C. Chen , Y.‐Q. Lei , L.‐F. Fang , B.‐K. Zhu , H. Matsuyama , J. Colloid Interface Sci. 2021, 584, 225. 33069021 10.1016/j.jcis.2020.09.041

[122] D. M. Pardhi , D. Şen Karaman , J. Timonen , W. Wu , Q. Zhang , S. Satija , M. Mehta , N. Charbe , P. A. McCarron , M. M. Tambuwala , H. A. Bakshi , P. Negi , A. A. Aljabali , K. Dua , D. K. Chellappan , A. Behera , K. Pathak , R. B. Watharkar , J. Rautio , J. M. Rosenholm , Int. J. Pharm. 2020, 586, 119531. 32540348 10.1016/j.ijpharm.2020.119531

[123] X.‐J. Yang , L.‐T. Geng , X.‐Y. Xu , X.‐Y. Shen , S. Sheng , F.‐A. Wu , J. Wang , Agronomy 2021, 11, 1159.

[124] Y. Zhang , W. Xiu , S. Gan , J. Shan , S. Ren , L. Yuwen , L. Weng , Z. Teng , L. Wang , Front. Bioeng. Biotechnol. 2019, 7, 218. 31552242 10.3389/fbioe.2019.00218 PMC6746923

[125] H. Le , C. Arnoult , E. Dé , D. Schapman , L. Galas , D. Le Cerf , C. Karakasyan , Biomacromolecules 2021, 22, 1639. 33709706 10.1021/acs.biomac.1c00082

[126] K. Ivanova , A. Ivanova , E. Ramon , J. Hoyo , S. Sanchez‐Gomez , T. Tzanov , ACS Appl. Mater. Interfaces 2020, 12, 35918. 32672937 10.1021/acsami.0c09364 PMC7497629

[127] J. C. Gutiérrez‐Santana , J. D. Toscano‐Garibay , M. López‐López , V. R. Coria‐Jiménez , Enferm. Infecc. Microbiol. Clin. Engl. Ed. 2020, 38, 331. 31948707 10.1016/j.eimc.2019.12.004

[128] D. Li , B. Zhou , B. Lv , J. Chem. 2020, 2020, 6578579.

[129] K. Urmann , J. Modrejewski , T. Scheper , J. Walter , BioNanomaterials 2016, 18, 20160012.

[130] J. Mehta , B. Van Dorst , E. Rouah‐Martin , W. Herrebout , M.‐L. Scippo , R. Blust , J. Robbens , J. Biotechnol. 2011, 155, 361. 21839787 10.1016/j.jbiotec.2011.06.043

[131] Z. Geng , L. Wang , K. Liu , J. Liu , W. Tan , Angew. Chem. Int. Ed. 2021, 60, 15459. 10.1002/anie.202102631 33904236

[132] Z. Geng , Z. Cao , R. Liu , K. Liu , J. Liu , W. Tan , Nat. Commun. 2021, 12, 6584. 34782610 10.1038/s41467-021-26956-8 PMC8593157

[133] K. M. Song , S. Lee , C. Ban , Sensors 2012, 12, 612. 22368488 10.3390/s120100612 PMC3279232

[134] H. Kaur , J. G. Bruno , A. Kumar , T. K. Sharma , Theranostics 2018, 8, 4016. 30128033 10.7150/thno.25958 PMC6096388

[135] S. Afrasiabi , R. Raoofian , M. Tabarzad , A. Bahador , J. Biomed. Sci. 2020, 27, 27. 31900238 10.1186/s12929-019-0611-0 PMC6941257

[136] A. Davydova , M. Vorobjeva , D. Pyshnyi , S. Altman , V. Vlassov , A. Venyaminova , Crit. Rev. Microbiol. 2016, 42, 847. 26258445 10.3109/1040841X.2015.1070115 PMC5022137

[137] I. Mohammed , D. Mohanty , D. G. Said , M. R. Barik , M. M. Reddy , A. Alsaadi , S. Das , H. S. Dua , R. Mittal , Br. J. Ophthalmol. 2021, 105, 1172. 32855162 10.1136/bjophthalmol-2020-316329

[138] J. H. Yeom , B. Lee , D. Kim , J. K. Lee , S. Kim , J. Bae , Y. Park , K. Lee , Biomaterials 2016, 104, 43. 27424215 10.1016/j.biomaterials.2016.07.009

[139] I. Ocsoy , S. Yusufbeyoglu , V. Yılmaz , E. S. McLamore , N. Ildız , A. Ülgen , Colloids Surf. B 2017, 159, 16. 10.1016/j.colsurfb.2017.07.056 28778062

[140] M. A Ocsoy , S. Yusufbeyoglu , N. Ildiz , A. Ulgen , I. Ocsoy , ACS Omega 2021, 6, 20637. 34396009 10.1021/acsomega.1c02832 PMC8359158

[141] S. Ucak , M. Sudagidan , B. A. Borsa , B. Mansuroglu , V. C. Ozalp , World J. Microbiol. Biotechnol. 2020, 36, 69. 32333113 10.1007/s11274-020-02845-y

[142] P. V. Baptista , M. P. McCusker , A. Carvalho , D. A. Ferreira , N. M. Mohan , M. Martins , A. R. Fernandes , Front. Microbiol. 2018, 9, 1441. 30013539 10.3389/fmicb.2018.01441 PMC6036605

[143] N .‐Y. Lee , W.‐C. Ko , P.‐R. Hsueh , Front. Pharmacol. 2019, 10, 1153. 31636564 10.3389/fphar.2019.01153 PMC6787836

[144] K.‐T. Kim , J.‐Y. Lee , D.‐D. Kim , I.‐S. Yoon , H.‐J. Cho , Pharmaceutics 2019, 11, 280.31197096

[145] M. R. Agel , E. Baghdan , S. R. Pinnapireddy , J. Lehmann , J. Schäfer , U. Bakowsky , Colloids Surf. B 2019, 178, 460. 10.1016/j.colsurfb.2019.03.027 30921681

[146] E. Baghdan , M. Raschpichler , W. Lutfi , S. R. Pinnapireddy , M. Pourasghar , J. Schäfer , M. Schneider , U. Bakowsky , Eur. J. Pharm. Biopharm. 2019, 139, 59. 30836179 10.1016/j.ejpb.2019.03.003

[147] J. B. Barhorst , R. Kubiak , Environ. Sci. Pollut. Res. 2009, 16, 582. 10.1007/s11356-009-0186-5 19479293

[148] K. Gopal , S. S. Tripathy , J. L. Bersillon , S. P. Dubey , J. Hazard. Mater. 2007, 140, 1. 17129670 10.1016/j.jhazmat.2006.10.063

[149] B. Legube , N. Karpel Vel Leitner , Catal. Today 1999, 53, 61.

[150] N. A. Napolitano , T. Mahapatra , W. Tang , Am. J. Infect. Control 2015, 43, 1342. 26277574 10.1016/j.ajic.2015.07.006

[151] Y. Liu , S. Dong , M. S. Kuhlenschmidt , T. B. Kuhlenschmidt , J. Drnevich , T. H. Nguyen , Water Res. 2015, 1, 188.

[152] M. Y. Song , J. Jurng , Y.‐K. Park , B. C. Kim , J. Hazard. Mater. 2016, 318, 247. 27427891 10.1016/j.jhazmat.2016.07.016

[153] Y. Wang , J. Wan , R. J. Miron , Y. Zhao , Y. Zhang , Nanoscale 2016, 8, 11143. 27180869 10.1039/c6nr01114d

[154] M. Seong , D. G. Lee , Curr. Microbiol. 2017, 74, 661. 28321528 10.1007/s00284-017-1235-9

[155] K. Zheng , M. I. Setyawati , D. T. Leong , J. Xie , Coord. Chem. Rev. 2018, 357, 1.

[156] X. Yuan , M. I. Setyawati , A. S. Tan , C. N. Ong , D. T. Leong , J. Xie , NPG Asia Mater. 2013, 5, e39.

[157] M. Yang , X. Chen , L. Zhu , S. Lin , C. Li , X. Li , K. Huang , W. Xu , ACS Appl. Mater. Interfaces 2021, 13, 38647. 34347427 10.1021/acsami.1c05751

[158] A. Aghebat Rafat , S. Sagredo , M. Thalhammer , F. C. Simmel , Nat. Chem. 2020, 12, 852. 32661410 10.1038/s41557-020-0504-6 PMC7116572

[159] A. Rajendran , M. Endo , Y. Katsuda , K. Hidaka , H. Sugiyama , J. Am. Chem. Soc. 2011, 133, 14488. 21859143 10.1021/ja204546h

[160] P. W. K. Rothemund , Nature 2006, 440, 297. 16541064 10.1038/nature04586

[161] S. Zhao , F. Duan , S. Liu , T. Wu , Y. Shang , R. Tian , J. Liu , Z. G. Wang , Q. Jiang , B. Ding , ACS Appl. Mater. Interfaces 2019, 11, 11112. 30874429 10.1021/acsami.8b21724

[162] N. C. Seeman , H. F. Sleiman , Nat. Rev. Mater. 2017, 3, 17068.

[163] Q. Jiang , S. Liu , J. Liu , Z.‐G. Wang , B. Ding , Adv. Mater. 2019, 31, 1804785. 10.1002/adma.201804785 30285296

[164] K. F. Wagenbauer , C. Sigl , H. Dietz , Nature 2017, 552, 78. 29219966 10.1038/nature24651

[165] C. Ducani , C. Kaul , M. Moche , W. M. Shih , B. Högberg , Nat. Methods 2013, 10, 647. 23727986 10.1038/nmeth.2503 PMC3843646

[166] Q. Zhang , Q. Jiang , N. Li , L. Dai , Q. Liu , L. Song , J. Wang , Y. Li , J. Tian , B. Ding , Y. Du , ACS Nano 2014, 8, 6633. 24963790 10.1021/nn502058j

[167] L. Song , Q. Jiang , J. Liu , N. Li , Q. Liu , L. Dai , Y. Gao , W. Liu , D. Liu , B. Ding , Nanoscale 2017, 9, 7750. 28581004 10.1039/c7nr02222k

[168] I. Mela , P. P. Vallejo‐Ramirez , S. Makarchuk , G. Christie , D. Bailey , R. M. Henderson , H. Sugiyama , M. Endo , C. F. Kaminski , Angew. Chem. Int. Ed. 2020, 59, 12698. 10.1002/anie.202002740 PMC7496991 32297692

[169] L. Mei , S. Zhu , Y. Liu , W. Yin , Z. Gu , Y. Zhao , Chem. Eng. J. 2021, 418, 129431.

[170] X. Meng , K. Fan , X. Yan , Sci. China Life Sci. 2019, 62, 1543. 31701405 10.1007/s11427-019-1557-8

[171] F. Cao , L. Zhang , H. Wang , Y. You , Y. Wang , N. Gao , J. Ren , X. Qu , Angew. Chem. Int. Ed. 2019, 58, 16236. 10.1002/anie.201908289 31456332

[172] Y. Zhang , Y. Jin , H. Cui , X. Yan , K. Fan , RSC Adv. 2020, 10, 10. 10.1039/c9ra09021e PMC9048033 35492517

[173] D. P. Cormode , L. Gao , H. Koo , Trends Biotechnol. 2018, 36, 15. 29102240 10.1016/j.tibtech.2017.09.006 PMC5738264

[174] J. Wu , S. Li , H. Wei , Chem. Commun. 2018, 54, 6520. 10.1039/C8CC01202D 29564455

[175] R. Zhang , Y. Zhou , X. Yan , K. Fan , Mikrochim. Acta 2019, 186, 782. 31729634 10.1007/s00604-019-3922-7

[176] H. Dong , Y. Fan , W. Zhang , N. Gu , Y. Zhang , Bioconjugate Chem. 2019, 30, 1273. 10.1021/acs.bioconjchem.9b00171 30966739

[177] X. Xiong , Y. Huang , C. Lin , X. Y. Liu , Y. Lin , Nanoscale 2019, 11, 22206. 31482920 10.1039/c9nr05054j

[178] D. Wang , D. Jana , Y. Zhao , Acc. Chem. Res. 2020, 53, 1389. 32597637 10.1021/acs.accounts.0c00268

[179] Q. Liu , A. Zhang , R. Wang , Q. Zhang , D. Cui , Nano‐Micro Lett. 2021, 13, 154. 10.1007/s40820-021-00674-8 PMC8271064 34241715

[180] L. Chen , S. Xing , Y. Lei , Q. Chen , Z. Zou , K. Quan , Z. Qing , J. Liu , R. Yang , Angew. Chem. Int. Ed. 2021, 60, 23534. 10.1002/anie.202107712 34378279

[181] D. Jana , D. Wang , A. K. Bindra , Y. Guo , J. Liu , Y. Zhao , ACS Nano 2021, 15, 7774. 33844517 10.1021/acsnano.1c01830

[182] Y. Sang , F. Cao , W. Li , L. Zhang , Y. You , Q. Deng , K. Dong , J. Ren , X. Qu , J. Am. Chem. Soc. 2020, 142, 5177. 32100536 10.1021/jacs.9b12873

[183] Q. Chen , Y. Liu , J. Liu , J. Liu , Chemistry 2020, 26, 16659. 33027544 10.1002/chem.202004133

[184] Y. Zhu , J. Zhang , J. Song , J. Yang , Z. Du , W. Zhao , H. Guo , C. Wen , Q. Li , X. Sui , L. Zhang , Adv. Funct. Mater. 2020, 30, 1905493.

[185] Z. Jiang , J. Guan , J. Qian , C. Zhan , Biomater. Sci. 2019, 7, 461. 30656305 10.1039/c8bm01340c

[186] K. Smerkova , K. Dolezelikova , L. Bozdechova , Z. Heger , L. Zurek , V. Adam , Wiley Interdiscip. Rev. Nanomed. Nanobiotechnol. 2020, 12, e1636. 32363802 10.1002/wnan.1636

[187] S. Preet , S. K. Pandey , K. Kaur , S. Chauhan , A. Saini , J. Drug Delivery Sci. Technol. 2019, 53, 101147.

[188] R. Sharma , R. Raghav , K. Priyanka , P. Rishi , S. Sharma , S. Srivastava , I. Verma , Sci. Rep. 2019, 9, 7866. 31133658 10.1038/s41598-019-44256-6 PMC6536545

[189] S. Joshi , R. Siddiqui , P. Sharma , R. Kumar , G. Verma , A. Saini , Sci. Rep. 2020, 10, 9441. 32523022 10.1038/s41598-020-66230-3 PMC7287048

[190] D. P. Singh , C. E. Herrera , B. Singh , S. Singh , R. K. Singh , R. Kumar , Mater. Sci. Eng. C 2018, 86, 173. 10.1016/j.msec.2018.01.004 29525091

[191] W. Choi , C. Battistella , N. C. Gianneschi , Biomater. Sci. 2021, 9, 653. 33300507 10.1039/d0bm01713b PMC9753762

[192] Q. Palomar , X. Xu , R. Selegård , D. Aili , Z. Zhang , Sens. Actuators B 2020, 325, 128789.

[193] Y. C. Chen , K. Y. A. Lin , C. C. Lin , T. Y. Lu , Y. H. Lin , C. H. Lin , K. F. Chen , Photochem. Photobiol. Sci. 2019, 18, 2442. 31384873 10.1039/c9pp00202b

[194] S. Yang , X. Han , Y. Yang , H. Qiao , Z. Yu , Y. Liu , J. Wang , T. Tang , ACS Appl. Mater. Interfaces 2018, 10, 14299. 29633833 10.1021/acsami.7b15678

[195] V. A. Spirescu , C. Chircov , A. M. Grumezescu , E. Andronescu , Polymers 2021, 13, 724. 33673451 10.3390/polym13050724 PMC7956825

[196] N. M. O. Andoy , K. Jeon , C. T. Kreis , R. M. A. Sullan , Adv. Funct. Mater. 2020, 30, 2004503.

[197] S. C. Park , C. Ko , H. Hyeon , M. K. Jang , D. Lee , ACS Appl. Mater. Interfaces 2020, 12, 54306. 33236874 10.1021/acsami.0c13083

[198] Z. Liu , K. Guo , N. Zhao , F.‐J. Xu , Sci. China Mater. 2019, 62, 1831.

[199] C. Wang , X. Gao , Z. Chen , Y. Chen , H. Chen , Polymers 2017, 9, 689. 30965987 10.3390/polym9120689 PMC6418682

[200] Y. Hu , Y. Li , F. J. Xu , Acc. Chem. Res. 2017, 50, 281. 28068064 10.1021/acs.accounts.6b00477

[201] R. Arshad , T. A. Tabish , M. H. Kiani , I. M. Ibrahim , G. Shahnaz , A. Rahdar , M. Kang , S. Pandey , Nanomaterials 2021, 11, 1086. 33922241 10.3390/nano11051086 PMC8146397

[202] Y. Liu , Z. Li , S. Zou , C. Lu , Y. Xiao , H. Bai , X. Zhang , H. Mu , X. Zhang , J. Duan , Int. J. Biol. Macromol. 2020, 155, 103. 32224180 10.1016/j.ijbiomac.2020.03.187

[203] H. Gu , P. L. Ho , E. Tong , L. Wang , B. Xu , Nano Lett. 2003, 3, 1261.

[204] K. H. Choi , H. J. Lee , B. J. Park , K. K. Wang , E. P. Shin , J. C. Park , Y. K. Kim , M. K. Oh , Y. R. Kim , Chem. Commun. 2012, 48, 4591. 10.1039/c2cc17766h 22473513

[205] S. K. Choi , A. Myc , J. E. Silpe , M. Sumit , P. T. Wong , K. McCarthy , A. M. Desai , T. P. Thomas , A. Kotlyar , M. M. Holl , B. G. Orr , J. R. Baker Jr. , ACS Nano 2013, 7, 214. 23259666 10.1021/nn3038995

[206] G. Qi , L.‐L. Li , F. Yu , H. Wang , ACS Appl. Mater. Interfaces 2013, 5, 10874. 24131516 10.1021/am403940d

[207] M. Chen , S. Xie , J. Wei , X. Song , Z. Ding , X. Li , ACS Appl. Mater. Interfaces 2018, 10, 36814. 30298721 10.1021/acsami.8b16092

[208] S. Palanikumar , L. Kannammal , B. Meenarathi , R. Anbarasan , Int. Nano Lett. 2014, 4, 104. 10.1016/j.ijbiomac.2014.03.012 24657379

[209] A. R. Chowdhuri , B. Das , A. Kumar , S. Tripathy , S. Roy , S. K. Sahu , Nanotechnology 2017, 28, 095102. 28139466 10.1088/1361-6528/aa57af

[210] Y. Wang , Q. Yuan , W. Feng , W. Pu , J. Ding , H. Zhang , X. Li , B. Yang , Q. Dai , L. Cheng , J. Wang , F. Sun , D. Zhang , J. Nanobiotechnol. 2019, 17, 103. 10.1186/s12951-019-0537-4 PMC6777033 31581948

[211] A. Allafchian , S. S. Hosseini , IET Nanobiotechnol. 2019, 13, 786. 31625518 10.1049/iet-nbt.2019.0146 PMC8676097

[212] R. Canaparo , F. Foglietta , F. Giuntini , C. Della Pepa , F. Dosio , L. Serpe , Molecules 2019, 24, 1991. 31137622 10.3390/molecules24101991 PMC6572634

[213] K. H. Markiewicz , I. Misztalewska‐Turkowicz , K. Niemirowicz , R. Bucki , A. M. Majcher , A. Z. Wilczewska , Arabian J. Chem. 2019, 12, 5187.

[214] J. K. Sahoo , S. K. Paikra , M. Mishra , H. Sahoo , J. Mol. Liq. 2019, 282, 428.

[215] J. M. V. Makabenta , A. Nabawy , C. H. Li , S. Schmidt‐Malan , R. Patel , V. M. Rotello , Nat. Rev. Microbiol. 2021, 19, 23. 32814862 10.1038/s41579-020-0420-1 PMC8559572

[216] US National Library of Medicine , ClinicalTrials.gov, https://clinicaltrials.gov/ct2/show/NCT02104245 (accessed: January, 2018).

